# Regression-type analysis for multivariate extreme values

**DOI:** 10.1007/s10687-022-00446-6

**Published:** 2022-10-21

**Authors:** Miguel de Carvalho, Alina Kumukova, Gonçalo dos Reis

**Affiliations:** 1 School of Mathematics, University of Edinburgh, Edinburgh, UK; 2 Maxwell Institute for Mathematical Sciences, School of Mathematics, University of Edinburgh, Edinburgh, UK; 3 Centro de Matemática e Aplicações (CMA), FCT, UNL, Caparica, Portugal

**Keywords:** Angular measure, Bernstein polynomials, Extreme value copula, Joint extremes, Multivariate extreme value distribution, Quantile regression, Statistics of extremes

## Abstract

This paper devises a regression-type model for the situation where both the response and covariates are extreme. The proposed approach is designed for the setting where the response and covariates are modeled as multivariate extreme values, and thus contrarily to standard regression methods it takes into account the key fact that the limiting distribution of suitably standardized componentwise maxima is an extreme value copula. An important target in the proposed framework is the regression manifold, which consists of a family of regression lines obeying the latter asymptotic result. To learn about the proposed model from data, we employ a Bernstein polynomial prior on the space of angular densities which leads to an induced prior on the space of regression manifolds. Numerical studies suggest a good performance of the proposed methods, and a finance real-data illustration reveals interesting aspects on the conditional risk of extreme losses in two leading international stock markets.

**Supplementary Information:**

The online version contains supplementary material available at 10.1007/s10687-022-00446-6.

## Introduction

Whereas classical statistical modeling is mostly concerned with inferences surrounding the bulk of a distribution, the field of statistics of extremes deals with the rather challenging situation of conducting inferences about the tail of a distribution. The behavior of extreme values in large samples is often mathematically tractable, and this tractability is often used to build sound statistical methods for modeling risk and extreme values. As an example of this asymptotic tractability, it is well known that if $Y_1, \dots , Y_n$ is a random sample with sample maximum $M_n = \max (Y_1, \dots , Y_n)$ and if there exist sequences $\{a_n > 0\}$ and $\{b_n\}$ such that $(M_n - b_n) / a_n {\overset{\mathrm {d}}{\rightarrow }}Z$, then *Z* follows a GEV (Generalized Extreme Value) distribution with location, scale, and shape parameters $\mu \in \mathbb {R}$, $\sigma > 0$, and $\xi \in \mathbb {R}$ respectively; see, for instance, Embrechts et al. (1997, Theorem 3.2.3). Details on the paradigm of statistics of extremes can be found in monographs (e.g. Coles 2001; Beirlant et al. 2004; de Haan and Ferreira 2006; Resnick 2007) as well as review papers (e.g. Davison and Huser 2015).

In this paper, we devise a regression-type method for the situation where both the response and the covariates are themselves extreme. Here and below, the expression “regression-type” is used to refer to the class of statistical models that relate the conditional quantiles of a response with covariates via a joint distribution—rather than by specifying a functional relation between response and covariate as, for example, in quantile regression (Koenker and Bassett 1978). An important result in the field of statistics of extremes—that will be fundamental for our developments—is that the properly standardized vector of block maxima converges in distribution to a so-called extreme value copula (Gudendorf and Segers 2010). Thus, a key target in the proposed framework is what we will refer to below as the regression manifold, that is, a family of regression lines that obeys the latter large sample result. Our methods thus take on board information on the dependence structure between the extreme values so to assess what effects an extreme value covariate can have on an extreme value response. To learn about the proposed model from data, we develop a prior in the space of regression manifolds by resorting to a flexible Bernstein polynomial prior on the space of angular densities as recently proposed by Hanson et al. (2017).

Our approach contributes to the literature on conditional modeling given large observed values (e.g. Wang and Stoev 2011; Cooley et al. 2012), nonetheless, our focus differs from the latter papers in a number of important ways as we describe next. The main difference is that, as anticipated above, here the focus is on devising a regression framework for extreme covariates and extreme responses, whereas the latter papers focus mainly on using the conditional density as a way to make probabilistic statements about the likelihood of an extreme given the occurrence of another extreme. Conditional quantile estimation within the framework of multivariate extreme value distributions was first looked into by Cooley et al. (2012). Here, among other things, we aim to characterize the entire regression manifold, its cross sections as well as how extremal dependence affects its shape; additionally, we aim to tackle the nontrivial task of defining a prior on the space of regression manifolds. Since our main target of analysis is regression, our method has some links with statistical approaches for nonstationary extremes (e.g. Coles 2001, Sect. 6; Yee and Stephenson 2007; Eastoe and Tawn 2009; Wang and Tsai 2009; Katz 2013); the most elementary version of approaches for nonstationary extremes aims to learn about how the limiting law of a suitably standardized block maxima response ($S_\mathbf {x}$) changes according to a covariate $\mathbf {x} = (x_1, \dots , x_p)^{\mathrm {\scriptscriptstyle T}}$, via the specification

1 $$\begin{aligned} (S \mid \mathbf {X} = \mathbf {x}) \sim \text {GEV}(\mu _{\mathbf {x}}, \sigma _{\mathbf {x}}, \xi _{\mathbf {x}}). \end{aligned}$$

Since the approach in (1) is built from the univariate theory of extremes it is not tailored for conditioning on another variable being extreme as it fails to take on board information from the dependence structure between the extremes.

Additionally, the method proposed in this work is loosely related to quantile regression (Koenker and Bassett 1978), whose original version consists in modeling the conditional quantile of a response *Y* given a covariate $\mathbf {X} = (X_1, \dots , X_p)^{\mathrm {\scriptscriptstyle T}}$ in a linear fashion, that is

2 $$\begin{aligned} F^{-1}(q \mid \mathbf {x}) = \mathbf {x}^{\mathrm {\scriptscriptstyle T}} {\boldsymbol{\beta }}_{q}, \qquad 0< q < 1, \end{aligned}$$

where $F^{-1}(q \mid \mathbf {x}) = \inf \{y: F(y \mid \mathbf {x}) \ge q\}$ and $F(y \mid \mathbf {x})$ is the distribution function of $Y \mid \mathbf {X} = \mathbf {x}$. Versions of quantile regression that aim to equip (2) with the ability to extrapolate into the tail of *Y* are often known as extremal quantile regression methods (e.g. Chernozhukov 2005). While flexible and sturdy, such quantile regression-based approaches do not take into account information on the fact that the limiting joint distribution of suitably standardized componentwise maxima is an extreme value copula, and thus fail to be equipped with the ability to extrapolate into the joint tail. The approach proposed in this paper will take such knowledge on the limiting joint distribution into consideration and will assume a conditional law that stems from such knowledge—rather than imposing a linear specification as in (2); yet, the proposed approach is not to be seen as a competitor to quantile regression but rather as a method based on some loosely related principles and specific to the context where we have a block maxima response and a block maxima covariate.

The remainder of the paper unfolds as follows. In Sect. 2 we introduce the proposed model and Sect. 3 devises an approach for learning about it from data. Section 4 reports the main findings of a Monte Carlo simulation study. We showcase the proposed methodology in a real data application to stock market data in Sect. 5. Finally, in Sect. 6 we present closing remarks. Supporting technical details can be found in the Appendix, and further numerical experiments and other details are presented in the supplementary material.

## Modelling conditional multivariate extreme value distributions

### Background on multivariate extremes

Prior to introducing a regression of block maxima on block maxima we need to lay groundwork on multivariate extremes. Let $\{(\mathbf {X}_{i}, Y_{i})\}_{i=1}^n$ be a sequence of independent random vectors with unit Fréchet marginal distributions, i.e. $\exp (-1 / z)$, for $z>0$. In our setup, $Y_i$ should be understood as a response, whereas $\mathbf {X}_i = (X_{1, i}, \dots , X_{p, i})$ should be understood as a *p*-dimensional covariate. Let the componentwise block maxima be $\mathbf {M}_n= (M_{n, x_1}, \dots , M_{n, x_p}, M_{n, y})$ with $M_{n, y} = \max \{ Y_1,\dots ,Y_n \}$ and $M_{n, x_j} = \max (X_{j,1}, \dots , X_{j,n})$, for $j = 1, \dots , p$. Under this setup, it is well-known that the vector of normalized componentwise maxima $\mathbf {M}_n/n$ converges in distribution to a random vector $(\mathbf {X}, Y)$ which follows a multivariate extreme value distribution with the joint distribution function

3 $$\begin{aligned} G(\mathbf {x}, y) = \exp \{-V(\mathbf {x}, y)\}, \quad (\mathbf {x}, y) \in (0, \infty )^{p + 1}. \quad \end{aligned}$$

Here,

$$\begin{aligned} V(\mathbf {x},y) = d \int _{\mathrm {\Delta }_d} \max \left( \dfrac{w_1}{x_1}, \dots , \dfrac{w_p}{x_{p}}, \dfrac{w_{p + 1}}{y} \right) \, H( {\mathrm {d}}\mathbf {w}), \end{aligned}$$

is the exponent measure and $d = p + 1$; see, for instance, de Haan and Resnick (1977), Pickands (1981), and Coles (2001, Theorem 8.1). In addition, *H* is a parameter of the multivariate extreme value distribution *G* known as angular measure, which controls the dependence between the extreme values; specifically, *H* is a probability measure on the unit simplex $\mathrm {\Delta }_d = \{(w_1, \dots , w_d) \in [0,1]^d, \sum _{i=1}^d w_i = 1\} \subset \mathbb {R}^d$, and obeying the mean constraint

4 $$\begin{aligned} \int _{\mathrm {\Delta }_d} \mathbf {w} \, H({\mathrm {d}}\mathbf {w}) = \dfrac{1}{d} \mathbf {1}_d, \end{aligned}$$

where $\mathbf {1}_d$ is a vector of ones in $\mathbb {R}^d$. If *H* is absolutely continuous with respect to the Lebesgue measure then its density is given by the Radon–Nikodym derivative $h = {\mathrm {d}}H / {\mathrm {d}}\mathbf {w}$, for $\mathbf {w} \in \mathrm {\Delta }_d$.

### Regression manifold for conditional multivariate extreme value distributions

We are now ready to introduce our regression method. We define the regression manifold as the family of regression lines,

5 $$\begin{aligned} \mathscr {L} = \{L_q: 0< q < 1\} \quad \text {with} \quad L_q = \{y_{q\mid \mathbf {x}}: \mathbf {x} \in (0, \infty )^p\}, \end{aligned}$$

where

6 $$\begin{aligned} y_{q\mid \mathbf {x}} = \inf \left\{ y>0: G_{Y\mid \mathbf {X}}(y\mid \mathbf {x}) \ge q \right\} , \end{aligned}$$

is a conditional quantile of a multivariate extreme value distribution, with $q \in (0,1)$ and $\mathbf {x} \in (0, \infty )^p$, and $G_{Y|\mathbf {X}}(y \mid \mathbf {x})=\mathbb {P}( Y \le y \mid \mathbf {X}=\mathbf {x})$ is a conditional multivariate extreme value distribution function. The next proposition sheds light on sufficient conditions in order for the regression manifold in (5) to be smooth.

#### Proposition 1

Let $G_{Y|X}(y \mid x) = \mathbb {P}(Y \le y \mid X=x)$ be a conditional multivariate extreme value distribution function. Suppose that $y \mapsto G_{Y|\mathbf {X}}(y\mid \mathbf {x})$ is continuous and strictly increasing over $[a, b] \subset (0, \infty )$, and that $\mathbf {x} \mapsto G_{Y|\mathbf {X}}(y\mid \mathbf {x})$ is continuous over $\mathbb {R}^p$. Then, $y_{q|\mathbf {x}}$ is continuous with respect to the sup norm for all $(\mathbf {x}, q) \in \mathbb {R}^p \times [c_{\mathbf {x}}, d_{\mathbf {x}}]$, where $c_{\mathbf {x}} = G_{Y|\mathbf {X}}(a\mid \mathbf {x})$ and $d_{\mathbf {x}} = G_{Y|\mathbf {X}}(b\mid \mathbf {x})$.

#### Proof

Let $(\mathbf {x}_0, z_0)$ be a point in $\mathbb {R}^p \times [c_{\mathbf {x}_0}, d_{\mathbf {x}_0}]$. We aim to show that for every $\varepsilon > 0$,

7 $$\begin{aligned} y_{0} - \varepsilon< y_{q|\mathbf {x}}< y_{0} + \varepsilon , \qquad \text {whenever } \Vert (\mathbf {x}, q) - (\mathbf {x}_0, q_0)\Vert _{\infty } < \delta , \end{aligned}$$

where $y_0 := y_{q_0|\mathbf {x}_0}$ and $\Vert (\mathbf {x}, z)\Vert _{\infty } = \max \{|x_1|, \dots , |x_p|, |z|\}$ is the sup norm. In order to find $\delta > 0$ that works for (7) we start by recalling that since by assumption $y \mapsto G_{Y|\mathbf {X}}(y \mid \mathbf {x})$ is continuous and strictly increasing, then it can be shown after some calculations by following a standard argument by Apostol (1980, Theorem 3.10) that there exists $\delta _1 > 0$, such that whenever $q_0 - \delta _1< q < q_0 + \delta _1$ it holds that

8 $$\begin{aligned} G_{Y|\mathbf {X}}(y_{0} - \varepsilon \mid \mathbf {x}_0)< q < G_{Y|\mathbf {X}}(y_{0} + \varepsilon \mid \mathbf {x}_0), \end{aligned}$$

Indeed, to obtain (8) it suffices to take

$$\delta _1 = \min \{G_{Y|\mathbf {X}}(y_0 + \varepsilon \mid \mathbf {x}_0) - G_{Y|\mathbf {X}}(y_0 \mid \mathbf {x}_0), G_{Y|\mathbf {X}}(y_0 \mid \mathbf {x}_0) - G_{Y|\mathbf {X}}(y_0 - \varepsilon \mid \mathbf {x})\}.$$

Now, by assumption $\mathbf {x} \mapsto G_{Y|\mathbf {X}}(y \mid \mathbf {x})$ is continuous and hence there exists a neighborhood of $\mathbf {x}_0$ with radius $\delta _2 > 0$ such that for every $\mathbf {x}$ in that neighborhood it holds that $G_{Y|\mathbf {X}}(y_0 - \varepsilon \mid \mathbf {x})< q < G_{Y|\mathbf {X}}(y_0 + \varepsilon \mid \mathbf {x})$, as a consequence of (8). This then implies that $y_{0} - \varepsilon< y_{q|\mathbf {x}} < y_{0} + \varepsilon$, and hence (7) holds with $\delta = \min (\delta _1, \delta _2)$, from where the final result follows.

In higher dimensions $G_{Y \mid \mathbf {X}}$ can be expressed with the help of a joint multivariate extreme value density $g_{\mathbf {X},Y}$ and its expression has been derived by Stephenson and Tawn (2005). By applying Bayes’ theorem, we deduce $G_{Y \mid \mathbf {X}}(y \mid \mathbf {x}) = \int _0^y g_{Y \mid \mathbf {X}}(z \mid \mathbf {x}) \, {\mathrm {d}}z$ from $g_{\mathbf {X},Y}$ with $g_{Y|\mathbf {X}}$ given as follows:

9 $$\begin{aligned} g_{Y|\mathbf {X}}(y \mid \mathbf {x}) = \dfrac{ \exp \{ - V(\mathbf {x},y) \} \sum \limits _{i=1}^d \sum \limits _{j=1}^{n_{i}} (-1)^i \prod \limits _{\Lambda \in r_{ij}} V(\mathbf {x},y) }{ \sum \limits _{i=1}^d \sum \limits _{j=1}^{n_{i}} (-1)^i \int \limits _0^{\infty } \exp \{ - V(\mathbf {x},y) \} \prod \limits _{\Lambda \in r_{ij}} V_{\Lambda }(\mathbf {x},y) \, {\mathrm {d}}y }, \quad y, \mathbf {x} >0, \end{aligned}$$

where $V_{\Lambda }(\mathbf {x},y)$ corresponds to mixed partial derivative of the exponent measure $V(\mathbf {x},y)$ with respect to the *l*th components of $(\mathbf {x},y)$ such that $l \in \Lambda$, $n_{i}$ is the number of partitions of $\{1,\dots ,d\}$ of size $i=1,\dots ,d$, and $r_{ij}$ is the *j*th partition of $\{1,\dots ,d\}$ of size *i*, with $1 \le j \le n_{i}$.

In the particular case where we have a single covariate $(p=1)$, the regression manifold $\mathscr {L}$ in (5) can be derived using properties of bivariate copulas; see Appendix A. Accordingly, for an absolutely continuous angular measure *H* (with density *h*), it follows that

10 $$\begin{aligned} G_{Y \mid X}(y \mid x) = 2 \, { \exp (1 / x) G(x,y) } \int _{\omega (x, y)}^1 w h(w) \, {\mathrm {d}}w, \quad x,y>0, \end{aligned}$$

where $\omega (x, y) = x / (x + y)$, and $y_{q\mid x}$ is then calculated via (6).

**Fig. 1:**
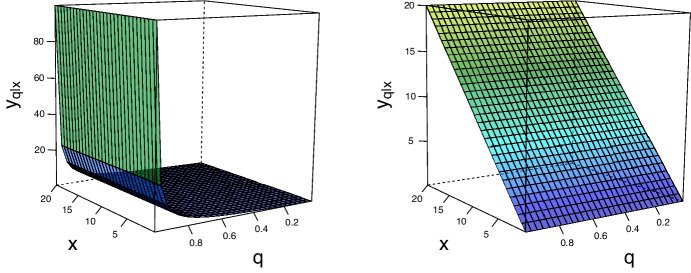
Regression manifolds for cases of complete independence (left) and perfect dependence (right)

We now derive regression manifolds $\mathscr {L}$ in (5) for the cases of independent and perfectly dependent extremes, which are depicted in Fig. 1. When extremes are independent, *H* assigns equal mass to the boundaries of the simplex, which also corresponds to asymptotic independence of $\mathbf {X}$ and *Y* (Hüsler and Li 2009), resulting in

11 $$\begin{aligned} L_q = \{ -1/\log q: \mathbf {x} \in (0, \infty )^p \}, \end{aligned}$$

with

$$\begin{aligned} G_{Y\mid \mathbf {X}}(y\mid \mathbf {x}) = \int _0^y \dfrac{\exp (- z^{-1} - x^{-1}_1 - \dots - x_{p}^{-1}) (-z^{-2}) \prod \limits _{j=1}^{p} (-x_j^{-2})}{ \exp (- x^{-1}_1 - \dots - x_{p}^{-1}) \prod \limits _{j=1}^{p} (-x_j^{-2})} \, {\mathrm {d}}z = \exp ({-1 / y}), \quad y > 0. \end{aligned}$$

When extremes are perfectly dependent, the angular measure *H* assigns all its mass to the barycenter of the simplex, $d^{-1}\mathbf {1}_d$, leading to $G(\mathbf {x},y) = \exp \{ - \max (x_1^{-1}, \dots , x_p^{-1}, y^{-1}) \}$. Taking derivatives of $G(\mathbf {x},y)$ in this case is non-trivial, and we replace the maximum function with a soft maximum (Cook 2011) so to obtain an approximation for the shape of the regression lines for perfectly dependent extremes. Thus, the soft maximum approximation for the regression lines for perfectly dependent extremes is

12 $$\begin{aligned} \tilde{L}_q = \{\min (x_1,\dots ,x_p): \mathbf {x} \in (0, \infty )^p\}. \end{aligned}$$

That is, regression lines for the case of perfectly dependent extremes do not depend on *q*. See Appendix B for the derivation, and Fig. 1 for a chart of its regression manifold.

We end this section with comments on properties of regression manifolds. Trivially, regression lines obey the standard properties of quantile functions (van der Vaart 1998, Chap. 21). Less trivial is however the fact that monotone regression dependence of bivariate extremes (Guillem 2000, Theorem 1) implies that regression lines $y_{q \mid x}$ in (6) are non-decreasing in *x*, for $p=1$, under some mild assumptions.

#### Proposition 2

Let $G_{Y|X}(y \mid x) = \mathbb {P}(Y \le y \mid X=x)$ be a conditional bivariate extreme value distribution function, which we assume to be jointly continuously differentiable and strictly increasing in *y* for any fixed $x \in (0,\infty )$. Then, the regression lines for bivariate extremes $(0,\infty )\ni x\mapsto y_{q\mid x}$ are non-decreasing for all $q\in (0,1)$.

#### Proof

Since $y\mapsto G_{Y \mid X} (y \mid x)$ is continuous (strictly increasing) for all $x \in (0,\infty )$, $y_{q\mid x}$ given by (6) is the solution to $G_{Y\mid X}(y\mid x) = q$ for a fixed $q \in (0,1)$. Then *y* satisfying $G_{Y\mid X}(y\mid x) = q$ is an implicit function of *x* parametrized by *q*. Under our assumptions we apply the implicit function theorem and calculate the derivative of $y_{q\mid x}$ with respect to *x* via

13 $$\begin{aligned} \frac{\partial }{\partial x}y_{q\mid x} = - \dfrac{\frac{\partial }{\partial x}G_{Y\mid X}(y\mid x)}{\frac{\partial }{\partial y}G_{Y\mid X}(y\mid x)}. \end{aligned}$$

Equation (13) combined with the monotone regression dependence property, i.e. $x\mapsto G_{Y\mid X}(y\mid x)$ is non-increasing for all $y \in (0,\infty )$ (Guillem 2000, Theorem 1), and the strict monotonicity of $y\mapsto G_{Y\mid X}(y\mid x)$ (increasing) for all $x \in (0,\infty )$ gives

$$\begin{aligned} \frac{\partial }{\partial x}y_{q\mid x} \ge 0. \end{aligned}$$

This completes the proof.

The assumption of $y\mapsto G_{Y \mid X} (y \mid x)$ being strictly increasing for all $x \in (0,\infty )$ is a mild assumption, which is satisfied by any bivariate GEV distribution that possesses an absolutely continuous angular measure with a positive density on (0, 1). This can be easily seen by noting that under those circumstances, it follows from (10) that

$$\begin{aligned} \frac{\partial G_{Y\mid X}}{\partial y} = 2 \exp (1/x) G(x,y) \left( \frac{2}{y^2} \int \limits _{\omega (x, y)}^1 w h(w) \, {\mathrm {d}}w \int \limits _0^{\omega (x, y)} (1-w) h(w) \, {\mathrm {d}}w + \omega ^3(x, y) \, h(\omega (x, y)) / x \right) > 0. \end{aligned}$$

Thus, monotonicity of regression manifold holds for Examples 1–3 below.

### Parametric instances of regression manifolds

**Fig. 2:**
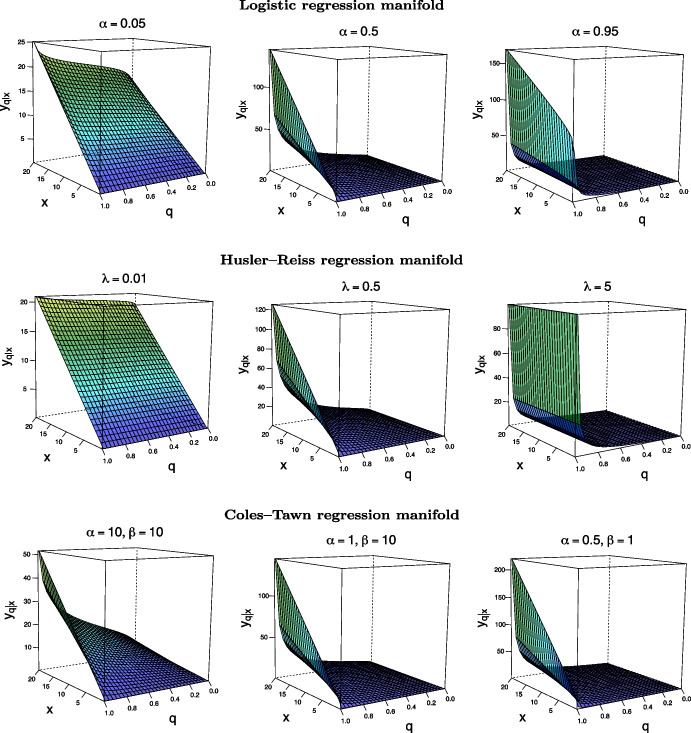
Regression manifold $\mathscr {L}$, as defined in (5), for bivariate Logistic, Husler–Reiss, and Coles–Tawn models (top to bottom) with strong dependence, intermediate and weak extremal dependence (left to right)

We now consider some parametric instances of regression manifolds as defined in (5). Charts of regression manifolds for these parametric examples are depicted in Fig. 2. In Appendix C, we show that for sufficiently large *x*, the following linear approximation holds for the regression manifold, $L_q = \{y_{q\mid x}: x \in (0, \infty )\}$, of the Logistic model from Example 1 with

14 $$\begin{aligned} y_{q\mid x} = \gamma _q + \beta _q x + o{(x)}. \end{aligned}$$

Here, $\gamma _q$ and $\beta _q$ are functions of both $\alpha$ and *q* (see Eqs. (24) and (25)), and *o*(*x*) is little-*o* of *x* in Bachmann–Landau notation; the numerical accuracy of this approximation is illustrated in the supplementary material.

#### Example 1

*(Logistic)* An instance of the Logistic regression manifold can be found in Fig. 2 (top). It stems from the Logistic bivariate extreme value distribution function given by

$$\begin{aligned} G(x,y) = \exp \{ - ( x^{-1/\alpha } + y^{-1/\alpha } )^{\alpha } \}, \quad x,y>0, \end{aligned}$$

where $\alpha \in (0,1]$ characterizes the dependence between extremes: the closer $\alpha$ to 0, the stronger the dependence, with the limit $\alpha \rightarrow 0$ corresponding to the case of perfect dependence. The conditional distribution of *Y* given *X* is

$$\begin{aligned} G_{Y\mid X}(y\mid x) = G(x,y) ( x^{-1/\alpha } + y^{-1/\alpha })^{\alpha - 1} x^{1-1/\alpha }\exp (1/x), \quad x,y > 0, \end{aligned}$$

thus leading to the following family of regression lines $L_q$ in (5) where

15 $$\begin{aligned} y_{q\mid x} = \left[ \left\{ \dfrac{1-\alpha }{\alpha } x W \left( \dfrac{\alpha }{1-\alpha } x^{-1} e^{ {\alpha }/(1-\alpha ) x^{-1}} q^{{\alpha }/(\alpha -1)} \right) \right\} ^{1/\alpha } - 1 \right] ^{-\alpha } x, \end{aligned}$$

and $x > 0$. Here, *W* is the so-called Lambert *W* function, that is, the multivalued analytic inverse of $f(z) = z \exp (z)$ with *z* denoting a real or complex number (Borwein and Lindstrom 2016); see the supplementary material for further details. As it can be seen from Fig. 2 (top), the regression lines obey what is claimed in Proposition 2 in the sense that $(0,\infty )\ni x\mapsto y_{q\mid x}$ are non-decreasing for all $q \in (0, 1)$.

#### Example 2

*(Husler–Reiss)* An instance of the Husler–Reiss regression manifold is depicted in Fig. 2 (middle). It follows from the Husler–Reiss bivariate extreme value distribution function which has the following form:

$$\begin{aligned} G(x,y) = \exp \left\{ - x^{-1} \Phi \left( \lambda + \dfrac{1}{2 \lambda } \log \dfrac{y}{x} \right) - y^{-1} \Phi \left( \lambda + \dfrac{1}{2 \lambda } \log \dfrac{x}{y} \right) \right\} , \quad x,y>0, \end{aligned}$$

where $\Phi$ is the standard Normal distribution function and $\lambda \in (0,\infty ]$ is the parameter regulating the dependence between extremes: $\lambda \rightarrow 0$ corresponds to perfect dependence and the limit case $\lambda \rightarrow \infty$ corresponds to complete independence. The family of regression lines $L_q$ in (5) for this model does not have explicit representations and is obtained using (6) with

$$\begin{aligned} G_{Y\mid X}(y\mid x)&= \left[ \Phi \left( \lambda + \dfrac{1}{2 \lambda } \log \dfrac{y}{x} \right) + { \dfrac{1}{2 \lambda } \phi \left( \lambda + \dfrac{1}{2\lambda } \log \dfrac{y}{x} \right) } - \dfrac{ x y^{-1}}{2\lambda } \phi \left( \lambda + \dfrac{1}{2 \lambda } \log \dfrac{x}{y} \right) \right] \\&\quad \times G(x,y) \exp (1 / x), \quad x,y > 0, \end{aligned}$$

where $\phi$ is the standard Normal density function.

#### Example 3

*(Coles–Tawn)* An instance of the Coles–Tawn regression manifold is depicted in Fig. 2 (bottom). It follows from the Coles–Tawn bivariate extreme value distribution function which has the following form:

$$\begin{aligned} G(x,y) = \exp [ - x^{-1} \{ 1 - \text {Be} ( q; \alpha + 1, \beta )\}- y^{-1} \text {Be}( q; \alpha , \beta + 1 ) ], \quad x,y>0, \end{aligned}$$

where $\text {Be}(q;a,b)$ is the cumulative distribution function of a Beta distribution with parameters $a,b > 0$, $q = \alpha y^{-1}/(\alpha y^{-1} + \beta x^{-1})$ and $\alpha ,\beta > 0$ are the parameters regulating dependence between extremes; the case $\alpha = \beta =0$ corresponds to complete independence, whereas $\alpha = \beta \rightarrow \infty$ corresponds to perfect dependence. For fixed $\alpha$ ($\beta$) the strength of dependence increases with $\beta$ ($\alpha$). The family of regression lines $L_q$ in (5) for this model does not have an explicit representation and is calculated using (6), for $x,y > 0$, with

$$\begin{aligned} G_{Y\mid X}(y\mid x) &= \Big [ 1 - \text {Be}\left( q; \alpha + 1, \beta \right)+ \dfrac{(\alpha + 1)\beta }{ \gamma } \text {be}\left( q; \alpha + 2, \beta + 1 \right) \\&- \dfrac{x}{y} \dfrac{\alpha (\beta + 1)}{\gamma } \text {be}\left( q; \alpha + 1, \beta + 2 \right) \Big ] G(x,y) \exp (1/x) , \end{aligned}$$

where $\text {be}(q;a,b)$ is the density function of the Beta distribution with parameters $a,b>0$ and $\gamma =(\alpha + \beta + 2)(\alpha + \beta + 1)$.

Section 2 introduced our key parameter of interest—regression manifolds for multivariate extreme values, i.e. $\mathscr {L}$ as in (5)—, it commented on some of its properties, and gave examples of parametric instances. Next, we discuss Bayesian inference for $\mathscr {L}$.

## Learning about regression manifolds via Bernstein polynomials

### Induced prior on the space of regression manifolds for $p=1$

In this section we discuss how to learn about regression manifolds from data. To achieve this, we resort to the Bayesian paradigm and will define an induced prior on the space of regression manifolds by resorting to a flexible prior on the space of all angular measures that was recently proposed by Hanson et al. (2017). To lay the groundwork, we start by defining the setup of interest. Let $\{(\mathbf {X}_i, Y_i)\}_{i=1}^n$ be a sequence of independent random vectors with unit Fréchet marginal distributions; define $R_i = Y_i + \sum _{j=1}^p X_{j,i}$ and $\mathbf {W}_i = (\mathbf {X}_i, Y_i)/ R_i$, known as the pseudo-angular decomposition of the observations. de Haan and Resnick (1977) showed the equivalence of the convergence of normalized componentwise maxima to *G* to the following weak convergence of measures

$$\begin{aligned} \mathbb {P}(\mathbf {W} \in \cdot \mid R > u) {\overset{\mathrm {d}}{\rightarrow }}H(\cdot ), \quad \text {as} \; u \rightarrow \infty . \end{aligned}$$

This means that when the radius *R* is sufficiently large, the pseudo-angles $\mathbf {W}$ are nearly independent of *R* and follow approximately a distribution associated with the angular measure *H*. Thus, to learn about $L_q$ in (5), we first learn about *H* based on $k = |\{\mathbf {W}_i: R_i > u, i = 1, \dots , n\}|$ exceedances above a large threshold *u*, where $k = o(n)$, with a methodology that we describe next.

Following Hanson et al. (2017), we model the angular density *h* via a Bernstein polynomial defined on the unit simplex $\mathrm {\Delta }_d$, and hence basis polynomials are Dirichlet densities. More precisely, our specification for the angular density is

16 $$\begin{aligned} h(\mathbf {w}) = {b(\mathbf {w}, J, {\boldsymbol{\pi }}_J)} := \sum \limits _{|{\boldsymbol{\alpha }}|=J} \pi _{{\boldsymbol{\alpha }}} \, \text {dir}_d(\mathbf {w}; {{\boldsymbol{\alpha }}}), \quad \end{aligned}$$

with $\mathbf {w} \in \mathrm {\Delta }_d$ and ${\boldsymbol{\pi }}_J = (\pi _{{\boldsymbol{\alpha }}}: |{\boldsymbol{\alpha }}| = J)$. Here, $\text {dir}_d$ is the density of a Dirichlet distribution supported on $\mathrm {\Delta }_d$, that is,

$$\begin{aligned} \text {dir}_d(\mathbf {w}; {\boldsymbol{\alpha }}) = \dfrac{\mathrm {\Gamma }(|{\boldsymbol{\alpha }}|)}{\prod \limits _{i=1}^d \mathrm {\Gamma }(\alpha _i)} \prod _{i=1}^d w_i^{\alpha _i-1}, \end{aligned}$$

where ${\boldsymbol{\alpha }}\in \mathbb {N}^d$ (with $\mathbb {N}:=\{1,2,3,\dots \}$), $|{\boldsymbol{\alpha }}|=\sum _{j=1}^d \alpha _j$, and $\mathrm {\Gamma }(z) = \int _0^\infty x^{z - 1} \exp (-x) \, {\mathrm {d}}x$ is the gamma function; finally in (16) the $\pi _{{\boldsymbol{\alpha }}} > 0$ are weights and $J \in \mathbb {N}$ controls the order of the resulting polynomial.

To ensure that the resulting $h(\mathbf {w})$ is a valid angular density (i.e. an actual density satisfying the moment constraint (4)), the weights must obey

17 $$\begin{aligned} \sum \limits _{|{\boldsymbol{\alpha }}|=J} \pi _{{\boldsymbol{\alpha }}} = 1, \qquad \sum \limits _{i=1}^{J-d+1} i \sum \limits _{|{\boldsymbol{\alpha }}|=J, \alpha _j=i} \pi _{{\boldsymbol{\alpha }}} = \dfrac{J}{d}, \end{aligned}$$

for $j=1,\dots ,d$. The normalization and mean constraints in (17) imply that there are $m - d$ parameters, where $m = {\binom{J-1}{d-1}}$ is the number of basis functions in (16); denote such free weights as $\{\pi _{{\boldsymbol{\alpha }}}: {\boldsymbol{\alpha }}\in \mathscr {F}\},$ where $\mathscr {F} = \{{\boldsymbol{\alpha }}\in \mathbb {N}^d, |{\boldsymbol{\alpha }}| = J, \text { and } {\boldsymbol{\alpha }}\not \in \{\mathbf {a}_1, \dots , \mathbf {a}_d\}\}$ with $\mathbf {a}_j$ being a *J*-vector of ones except element *i* is $J - d + 1$. Similarly to Hanson et al. (2017), we parametrize the free weights via a generalized logit transformation that implicitly defines the auxiliary parameters $\pi _{{\boldsymbol{\alpha }}}'$, that is,

18 $$\begin{aligned} \pi _{{\boldsymbol{\alpha }}} = \frac{\exp (\pi _{{\boldsymbol{\alpha }}}')}{d + \sum _{\tilde{\boldsymbol{\alpha }}\in \mathscr {F}}\exp (\pi _{\tilde{\boldsymbol{\alpha }}}')}. \end{aligned}$$

Now, to induce a prior in the space of regression manifolds we plug-in the angular density in (16) into (9); subsequent integration with respect to *y* and inversion of $G_{Y|\mathbf {X}}(y|\mathbf {x})$ leads to an induced prior on the space of regression lines $L_q$. In detail, to define a prior on the space of regression manifolds we proceed as follows. The Bernstein polynomial prior in (16) induces a prior on the space of regression lines $L_q = \{{y}_{q \mid x}: x \in (0,\infty )\}$, where ${y}_{q \mid x}$ is a solution to equation, ${G}_{Y\mid X} (y\mid x) = q$, for $q \in (0,1)$, where

19 $$\begin{aligned} \begin{aligned}&{G}_{Y\mid X} (y\mid x)\\ {}&= \dfrac{2}{J} \exp \Bigg \{-\dfrac{2}{J} \sum \limits _{|\alpha |=J} \pi _{{\boldsymbol{\alpha }}}[ \alpha _1 x^{-1} \{ 1 - \text {Be}(\omega (x, y);\alpha _1 + 1,\alpha _2)\} + \alpha _2 y^{-1} \text {Be}(\omega (x, y);\alpha _1,\alpha _2+1)] \Bigg \}\\ {}&\quad \times \sum \limits _{|{\boldsymbol{\alpha }}|=J} \pi _{{\boldsymbol{\alpha }}} \alpha _1 \{1 - \text {Be}(\omega (x, y);\alpha _1+1,\alpha _2) \} \exp (1/x), \end{aligned} \end{aligned}$$

where $\omega (x, y) = x / (x + y)$, for $x, y > 0$. Finally, to complete the model specification we set the following Dirichlet prior on the free parameters

$$\begin{aligned} p(\pi _{{\boldsymbol{\alpha }}}: {\boldsymbol{\alpha }}\in \mathscr {F}) \propto \text {dir}_d(\mathbf {w} \mid c \, \mathbf {1}_m) \prod _{j = 1}^d I\left\{ \sum \limits _{i=1}^{J-d+1} i \sum \limits _{|{\boldsymbol{\alpha }}|=J, \alpha _j=i} \pi _{{\boldsymbol{\alpha }}} = \dfrac{J}{d}\right\} , \end{aligned}$$

where *I* is the indicator function, which accordingly induces a prior on the auxiliary parameters $\pi _{{\boldsymbol{\alpha }}}'$ in (18).

### Induced prior on the space of regression manifolds for ${p\ge 1}$

This section will show how an approximation due to Cooley et al. (2012) can be used for conducting inference in the case $p \ge 2$; while the main focus will be on $p \ge 2$ the provided approximation works as well in the case $p = 1$, and thus it can be regarded as an alternative to the exact approach from Sect. 3.1.

When $p \ge 2$ we proceed as in Sect. 3.1, that is our induced prior in the space of regression lines is again induced by the Bernstein polynomial prior for the angular density in (16), and it follows by solving ${G}_{Y\mid \mathbf {X}} (y\mid \mathbf {x}) = q$, with $h(\mathbf {w})$ as in (16). The expression for the conditional multivariate extreme value distribution ${G}_{Y\mid \mathbf {X}} (y\mid \mathbf {x})$ for $p \ge 2$ is however not as manageable as the one in (19). We thus propose an approach for learning about the regression manifold $\mathscr {L}$, as defined in (5), via an approximation to the conditional multivariate GEV density. Let $\mathbf {u}=(\mathbf {x}, y) \in (0,\infty )^d$ and $\mathbf {u}(t)=(\mathbf {x}, t) \in (0,\infty )^d$ with $\Vert \mathbf {u} \Vert = y + \sum _{i=1}^p x_i > u$ for a large threshold *u*.

Then, following Cooley et al. (2012, Proposition 1) the conditional density of a multivariate extreme value distribution can be approximated, via a point process representation for extremes, as follows

20 $$\begin{aligned} g_{Y \mid \mathbf {X}} (y \mid \mathbf {x}) \approx \dfrac{\Vert \mathbf {u} \Vert ^{-d-1} h(\mathbf {u}/\Vert \mathbf {u} \Vert )}{\int _0^{\infty } \Vert \mathbf {u}(t) \Vert ^{-d-1} h(\mathbf {u}(t)/\Vert \mathbf {u}(t) \Vert ) \, {\mathrm {d}}t}. \end{aligned}$$

**Fig. 3:**
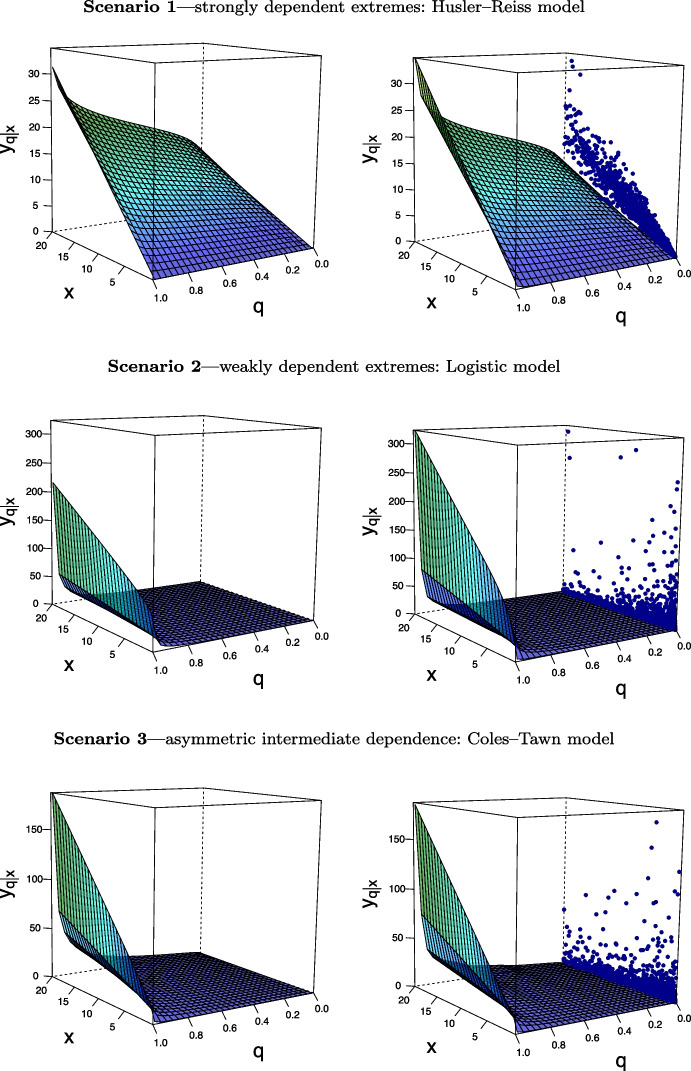
True regression manifold $\mathscr {L}$, as defined in (5), along with its posterior mean estimate obtained using the methods from Sect. 3 for Husler–Reiss, Logistic, and Coles–Tawn bivariate extreme value models (top to bottom) on a single-run experiment. Simulated data are overlaid on one of the faces of the box

An induced prior for *g* can be devised by plugging the approximation in (20) with the specification from Sect. 3.1, which leads to the following prior for the conditional multivariate extreme value distribution function,

21 $$\begin{aligned} G_{Y \mid \mathbf {X}} (y \mid \mathbf {x}) \approx \dfrac{\sum \limits _{|\alpha |=J} \pi _{{\boldsymbol{\alpha }}} / B_{{\boldsymbol{\alpha }}} \prod \limits _{i=1}^p x_i^{\alpha _i-1} \int _0^y t^{\alpha _d-1} \Vert \mathbf {u}(t) \Vert ^{-J-1} \, {\mathrm {d}}t}{\sum \limits _{|\alpha |=J} \pi _{{\boldsymbol{\alpha }}} / B_{{\boldsymbol{\alpha }}} \prod \limits _{i=1}^p x_i^{\alpha _i-1} \int _0^{\infty } t^{\alpha _d-1} \Vert \mathbf {u}(t) \Vert ^{-J-1} \, {\mathrm {d}}t}, \end{aligned}$$

where $B_{{\boldsymbol{\alpha }}} = \prod _{i=1}^d \mathrm {\Gamma }(\alpha _i) / \mathrm {\Gamma }(|{\boldsymbol{\alpha }}|)$ is the multivariate beta function. Hence, we can learn about the regression manifold $\mathscr {L}$ by estimating $\pi _{{\boldsymbol{\alpha }}}$ as described in Sect. 3.1, that is, by plugging in the Bernstein polynomial estimates (16) into the approximation (20) and numerically inverting (21) with respect to *y*. This strategy is illustrated numerically in the supplementary material.

## Simulation study

### Preliminary experiments

We study the finite sample performance of the proposed methods under three data generating scenarios that were introduced in Sect. 2; see Examples 1–3. Specifically, we simulate data as follows:

- **Scenario 1**—strongly dependent extremes: Husler–Reiss model with $\lambda =0.1$.
- **Scenario 2**—weakly dependent extremes: Logistic model with $\alpha =0.9$.
- **Scenario 3**—asymmetric intermediate dependence: Coles–Tawn model with $\alpha = 0.5$, $\beta = 100$.

For now we focus on illustrating the methods in a single-run experiment; a Monte Carlo simulation study will be reported in Sect. 4.2. To illustrate how the resulting estimates compare with the true regression lines on a one-shot experiment, in each scenario we generate $n=5\,000$ samples $\{(\mathcal {X}_i,\mathcal {Y}_i)\}_{i=1}^n$. For the analysis we use observations for which $\widehat{X}_i+\widehat{Y}_i > u$, where *u* is the $98\%$ quantile; here, the raw data are transformed to unit Fréchet margins via the transformation $(\widehat{X}_i, \widehat{Y}_i) = (- 1 / \log \{\widehat{F}_\mathcal {X}(\mathcal {X}_i)\}, - 1 / \log \{\widehat{F}_\mathcal {Y}(\mathcal {Y}_i)\}),$ where $\widehat{F}_\mathcal {X}$ and $\widehat{F}_\mathcal {Y}$ respectively denote the empirical distribution functions (normalized by $n + 1$ rather than by *n* to avoid division by zero). To learn about regression lines from data, we employ a standard componentwise adaptive Markov Chain Monte Carlo (MCMC) (Haario et al. 2005) with a Dirichlet prior, Dirichlet$(10^{-4} \mathbf {1}_k)$, defined on a generalized logit transformation of weights $\pi _{{\boldsymbol{\alpha }}}$. The length of each MCMC chain is $10\,000$ with a burn-in period of $4\,000$.

**Fig. 4:**
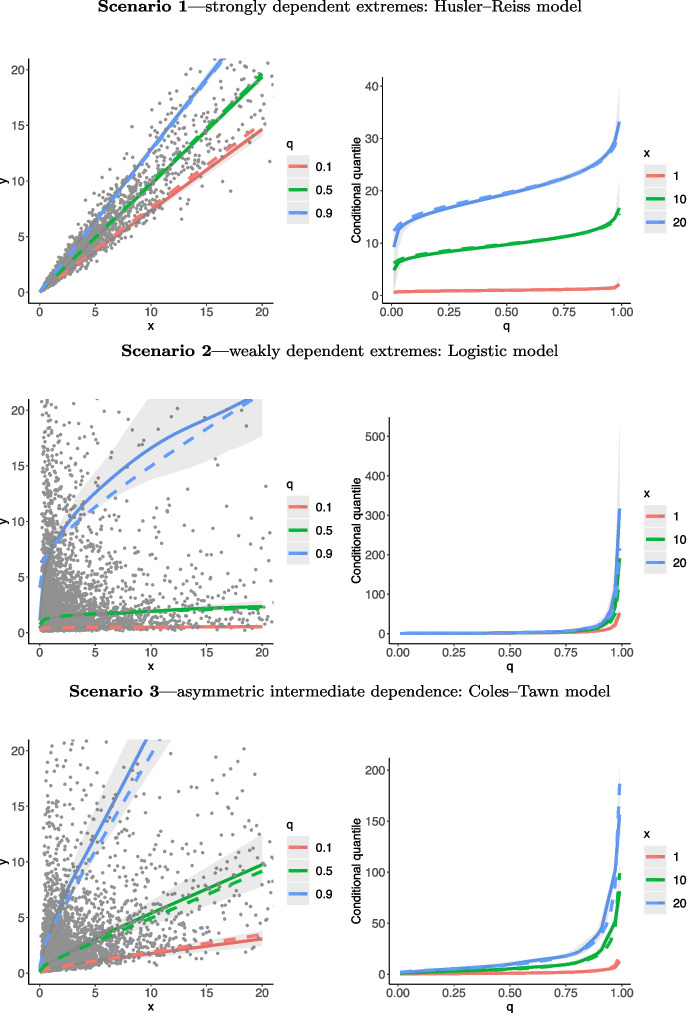
Posterior mean regression lines $L_q$ for $q \in \{0.1,0.5,0.9\}$ and $x \in (0,20]$ (left) and conditional quantile curves $\{y_{q \mid x}:q\in (0,1)\}$ along with credible bands, for $x = \{1,10,20\}$ (right) for Husler–Reiss, Logistic, and Coles–Tawn bivariate extreme value models (top to bottom) on a single-run experiment. The dashed lines represent the true

In Fig. 3 we plot true and estimated regression manifolds under the three scenarios above over the range $(x ,y) \in (0,20]\times (0,20]$, where 20 corresponds to the $95\%$ quantile of the unit Fréchet marginal distributions. Figure 3 shows that, for these one shot experiments, the proposed methods recover well the shape of $\mathscr {L}$ for all three cases, although as expected the fits are slightly less accurate for *q* closer to 0 and 1. To have a closer look into the outputs from these numerical experiments, we depict in Fig. 4 cross sections of the angular manifold, over *q* and over *x*, thus leading to regression lines and conditional quantiles for the Husler–Reiss (top), Logistic (central), and Coles–Tawn (bottom) models. Once more, we see that the fits are fairly reasonable overall although a bit more of bias is visible for *q* closer to 0 and 1, as can be seen in the charts of the conditional quantile curves. Interestingly, it can also be noticed in Fig. 4 that regression lines are approximately linear for the Logistic model, and we prove that this indeed is the case for large *x*; see Appendix C.

Figure 4 also anticipates a feature that we will revisit in Sect. 4.2, i.e. that the more the regression lines change with *q*, the larger is the uncertainty; this explains why the bands of the regression lines for Scenario 3 are wide, while those of Scenario 1 are narrow. Similarly, the more abruptly the regression lines change with *q*, the larger is the uncertainty. For example, for Scenario 2 it can be noticed that the true regression line for $q = 0.9$ attains values much larger than the cross sections $q = 0.1, 0.45, 0.55$, and indeed the bands are wider for $q = 0.9$.

Motivated by the computational experience of one of the authors on a recent paper (Galasso et al. 2022), we have opted not to set a prior on *J*. This allows for any user to replicate our Monte Carlo simulation studies, and not just those who have access to Google Cloud, Amazon Web Services or a University server, as our methods are easy to code and can be readily implemented in a standard desktop machine; for example, the full Monte Carlo simulation study reported in Sect. 4.2 takes around 5 hours to run on a 3,2 GHz 6-Core Intel Core i7 (64 GB) machine. Hence, rather than setting a prior on *J*, we have opted for the computationally appealing and effective approach of Hanson et al. (2017) where *J* is chosen to be maximized subject to the number of basis functions being smaller or equal to the number of pseudo-angles. Part of the rationale for setting a large *J* is the well-known fact that, as $J \rightarrow \infty$, Bernstein polynomials can uniformly approximate any continuous and bounded density on the unit interval (Altomare and Campiti 1994, p. 333) and multivariate Bernstein polynomials obey a similar property (Barrientos et al. 2015, Sect. 4.1). As shown in Sect. 3 of the supplementary material, this strategy of choosing *J* of Hanson et al. (2017) leads to comparable performance, if not superior, with respect to the likelihood ratio changepoint approach of Guan (2016).

**Fig. 5:**
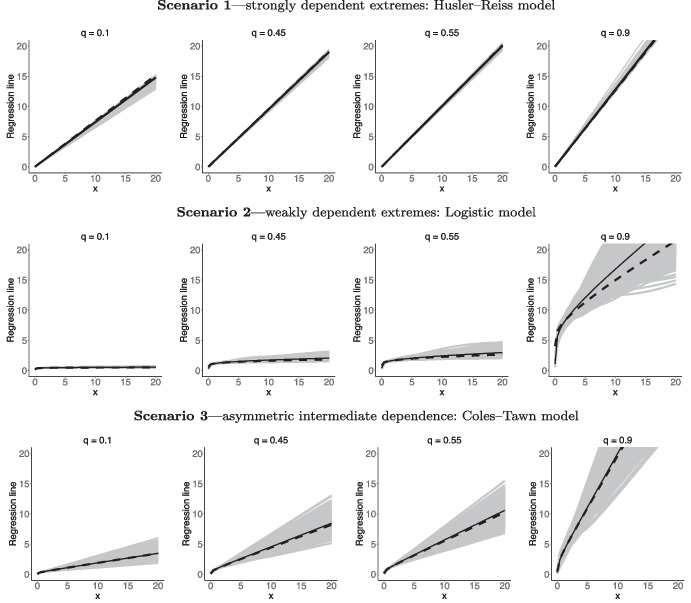
Posterior mean regression lines $L_q$ for $q \in \{0.1,0.45,0.55,0.9\}$ and $x \in (0,20]$ for each of the 500 Monte Carlo samples (gray lines) plotted against the true (dashed line) for Husler–Reiss, Logistic, and Coles–Tawn bivariate extreme value models (top to bottom). The solid black line represents the Monte Carlo mean

### Monte Carlo simulations

To conduct a simulation study we generate 500 Monte Carlo samples of size $n=5\,000$ thresholded at the $98\%$ quantile for the three scenarios described in Sect. 4.1. We use the MCMC algorithm as described in Sect. 4.1 with the same prior specification. The performance of our methods will be visualized via a comparison of posterior mean estimates of the regression lines with the true regression lines $L_q$ for a few fixed $q \in (0,1)$. We focus on the region $x \in (0,20]$ as the bivariate extreme value concentrates most of its mass (at least 90%) in the set $(0,20]\times (0,20]$.

The regression lines corresponding to the described scenarios are shown in Fig. 5. Figure 5 shows that the model fits the data for all scenarios reasonably well but again it can be noticed that the fit is slightly less accurate in Scenario 3 for $q = 0.9$. As mentioned in Sect. 4.1, our computational experience with the method leads us to believe that this is due to how suddenly the cross section changes for higher *q*, as indeed the true regression line for $q = 0.9$ attains values much larger than those of $q = 0.1, 0.45$, and 0.55. Interestingly, while the Monte Carlo means for Scenario 1 are in line with the true there is some asymmetry visible in the rare outlying fits in Fig. 5 though their frequency is not sufficient to shift the Monte Carlo means away from the true.

In addition to the numerical experiments reported above we have conducted a battery of other studies. Section 3 of the supplementary material includes a sensitivity analysis on the selected threshold as well as a similar Monte Carlo study for $n = 10\,000$. The results in the supplementary material suggest similar findings when the $95\%$ quantile is used for thresholding the data, but with the asymmetry effect mentioned above being slightly attenuated; one can also notice in that additional study a moderate improvement in the fits when $n = 10\,000$. Beyond the simulation scenarios examined above, we also report in the supplementary material an additional scenario with a discrete angular measure; despite the fact that our prior is defined on the space of continuous angular measures the results available from the supplementary material indicate a satisfactory performance even on the latter case.

## Real data illustration

### Data, preprocessing, and applied rationale for the analysis

#### Description and scope

We now apply the proposed method to two of the world’s biggest stock markets—the NASDAQ (National Association of Securities Dealers Automated Quotations) and the NYSE (New York Stock Exchange). According to the Statistics Portal of the World Federation of Exchanges (https://statistics.world-exchanges.org), the total equity market capitalization of NASDAQ and NYSE are respectively 20.99 and 24.67 trillion US$, as of April 2021, thus illustrating well the scale of these players in the worldwide stock-exchange industry. The data, available from the R package DATAstudio, were gathered from Yahoo Finance, and consist of daily closing prices of the NASDAQ and NYSE composite indices over the period from February 5, 1971 to June 9, 2021. A key goal of the analysis to be reported in Sect. 5.2 is to showcase how our model can be used to learn about the conditional risk of an extreme loss in the NYSE, given an extreme loss in the NASDAQ. Before we present the bulk of the conditional risk analysis via our methods we first offer below some remarks on the data.

**Fig. 6:**
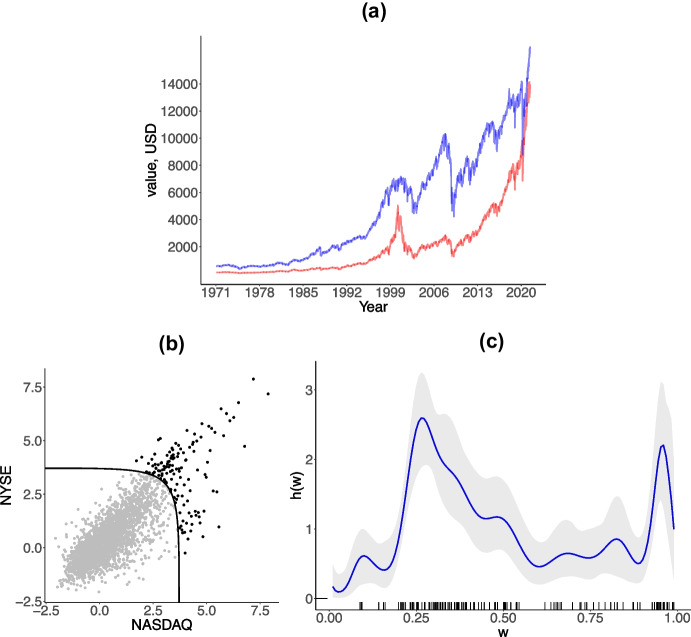
(**a**) NASDAQ (red) and NYSE (blue) composite indices. (**b**) Scatterplot of negative log returns of NASDAQ and NYSE composite indices converted to unit Fréchet margins; the solid line corresponds to the boundary threshold in the log-log scale, with both axes being logarithmic. (**c**) Angular density estimate with $95\%$ credible band along with a rug of pseudo-angles

We will focus on negative log returns, which can be regarded as a proxy for losses and which consist of first differences of prices on a log-scale; the resulting sequence of *n* componentwise weekly maxima losses for NASDAQ and NYSE is denoted below as $\{(\mathcal {X}_i, \mathcal {Y}_i)\}_{i = 1}^n$. The sample period under analysis is sufficiently broad to cover a variety of major downturns and selloffs including, for example, those related with the 2007–2010 subprime mortgage crisis, the ongoing China–US trade war, and with the 2020 COVID-19 pandemic. Similarly to Sect. 4, we convert negative log returns to unit Fréchet margins via the transformation $(\widehat{X}_i, \widehat{Y}_i) = (- 1 / \log \{\widehat{F}_\mathcal {X}(\mathcal {X}_i)\}, - 1 / \log \{\widehat{F}_\mathcal {Y}(\mathcal {Y}_i)\}),$ where $\widehat{F}_\mathcal {X}$ and $\widehat{F}_\mathcal {Y}$ respectively denote the empirical distribution functions (normalized by $n + 1$ rather than by *n* to avoid division by zero) of negative log returns for NASDAQ ($\mathcal {X}$) and NYSE ($\mathcal {Y}$); the supplementary material includes the reverse analysis that swaps the roles of NASDAQ and NYSE (i.e. NASDAQ becomes $\mathcal {Y}$ and NYSE becomes $\mathcal {X}$). To validate the use of our model we further test negative log returns on NYSE and NASDAQ for multivariate regular variation (MRV) following methodology introduced by Einmahl et al. (2021). The results of the conducted analysis are presented in Sect. 4 of the supplementary material and suggest that at a $5\%$ significance level there is no evidence to reject that the pair (NYSE, NASDAQ) follows a MRV distribution for a broad range of thresholds.

#### Visualization

The raw data and resulting preprocessed data are depicted in Fig. 6. As can be seen from the latter figure the composite indices exhibit a similar dynamics reacting to different economic shocks (9/11 attacks, 2001; 2008 financial crisis; China-US trade war started in 2018) alike. Also, as can be seen from Fig. 6, the shape of the scatterplot of the negative log returns brought to unit Fréchet margins in log-log scale above the boundary threshold evidences intermediate level of extremal dependence between negative log returns.

Before we focus on conditional risk (Sect. 5.2), we start by fitting the angular density via the Bernstein polynomial-based approach from Sect. 3.1. We follow essentially the same settings for prior specification and MCMC as in Sect. 4, threshold the unit Fréchet data at its 95% quantile, set the number of basis functions using once more the approach of Hanson et al. (2017), and run an MCMC chain of length $25\,000$ with a burn-in period of $10\,000$. The specified chain has the multivariate effective sample size of $1\,726\,657$. The obtained fit for the angular density is reported in Fig. 6 (right). As is illustrated by this plot most of the observed pseudo-angles lie closer to the middle of the interval (0, 1) and the estimate resembles a bell-shaped right-skewed density which suggests there is an asymmetric intermediate dependence between extremal losses on NASDAQ and NYSE composite indices. Such asymmetry suggests a tendency for the losses of the NYSE to be more extreme than those of NASDAQ, when both are extreme.

**Table 1:** Predicted $75\%$, $90\%$ and $95\%$ quantiles of losses on NYSE evaluated for $1\%$, $2\%$ and $3\%$ weekly maxima losses on NASDAQ, with $95\%$ credible intervals in brackets; negative log-returns used as proxy for losses

NYSE	NASDAQ		
	0.01	0.02	0.03
$75\%$	0.0129	0.0194	0.0266
	(0.0125, 0.0134)	(0.0187, 0.0201)	(0.0259, 0.0274)
$90\%$	0.0156	0.0233	0.0315
	(0.0153, 0.0159)	(0.0229, 0.0237)	(0.0309, 0.0319)
$95\%$	0.0171	0.0249	0.0333
	(0.0166, 0.0177)	(0.0245, 0.0255)	(0.0328, 0.0339)

### Modeling conditional risk via regression manifolds

**Fig. 7:**
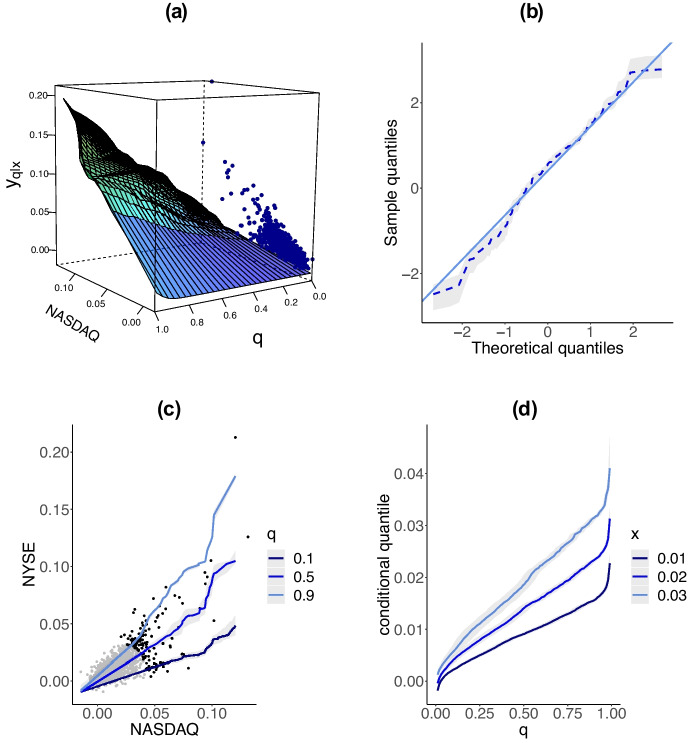
(**a**) Posterior mean regression manifold on the original scale for NYSE given NASDAQ along with joint negative log returns overlaid on one of the faces of the box. (**b**) QQ-plot of randomized quantile residuals; the dashed line represents the posterior mean plotted along with credible bands. (**c**) Posterior mean regression lines $L_q$ for $q\in \{0.1,0.5,0.9\}$ for NYSE given NASDAQ along with $95\%$ credible bands and plotted against joint negative log returns. (**d**) Posterior mean conditional quantile curves $\{y_{q \mid x}:q\in (0,1)\}$ of negative log returns on NYSE for $x\in \{0.01,0.02,0.03\}$, along with $95\%$ credible bands, corresponding to negative log returns on NASDAQ in the original margins

This section presents the bulk of the conditional risk analysis. Specifically, we will show how the regression manifold can be used to model the conditional risk of an extreme loss on the NYSE, given an extreme loss on the NASDAQ. Figure 7a represents the resulting estimates of the regression manifold on the original scale together with cross-sections in *q* and *x*, respectively in (b) and (d) for negative log-returns on NASDAQ and NYSE composite indices in the original margins. Having evaluated the regression manifold, we are now ready to extract from its cross sections information on by how much the NYSE can plummet, conditionally on the NASDAQ plummeting. To examine this, we report in Table 1 predicted $75\%$, $90\%$ and $95\%$ quantiles of losses on NYSE evaluated for $1\%$, $2\%$ and $3\%$ weekly maxima losses on NASDAQ. This table follows from the regression manifold, and its interpretation is as follows. First, from a qualitative viewpoint, Table 1 indicates that conditionally on the NASDAQ plummeting, the NYSE tends to plummet reasonably by the same amount. Second—and more interesting from a financial outlook—are the quantitative claims that can be made from the analysis. For example, Table 1 indicates that whenever there is a $1\%$ weekly maximum loss on the NASDAQ, only in 5% of the times we expect to suffer a loss in the NYSE above $1.71\%$. As another example, Table 1 indicates that whenever there is a $3\%$ weekly loss on the NASDAQ, only in 5% of the times we expect to suffer a loss in the NYSE above $3.33\%$.

As can be seen from Fig. 7 the regression manifold is highly non-linear and the regression lines on the middle graph substantially differ from those corresponding to independence and tend to be closer to the identity line. Moreover, the cross-sections for different values of *x* reveal considerable variation in quantiles of *y* supporting the conclusion about presence of the dependence between negative log-returns. To assess the quality of the fitted regression manifold we depict in Fig. 6b a QQ-plot of a version of Dunn and Smyth (1996) randomized quantile residuals adapted to our model, defined as $\varepsilon _{i} = \Phi ^{-1}(G_{H}(Y_i \mid X_i))$, for $Y_i + X_i > u$, with *u* denoting the 95% quantile of the pseudo-radius. The latter chart depicts randomized quantile residuals against the theoretical standard Normal quantiles, and it suggests an acceptably good fit of the proposed model. Beyond the results shown above we have conducted a variety of other empirical analysis which are available from the supplementary material (Sect. 5). In particular, we report in the supplementary material the fitted regression manifold that results from combining our random Bernstein model with the approximation from Sect. 3.2. As expected, the results are tantamount to the ones presented herein.

## Closing remarks

We propose a regression-type model for the setup where both the response and the covariate are extreme. The modeling starting point is the result that the limiting behavior of the vector of properly standardized componentwise maxima is given by a multivariate extreme value distribution. Conceptually, the model is then constructed in a similar fashion as in quantile regression, that is, by assessing how the conditional quantile of the response reacts to changes in the covariate while it takes into account the latter asymptotic result. An important target in the proposed framework is the regression manifold, which consists of a family of regression lines obeying the proviso of multivariate extreme value theory. A Bernstein polynomial prior on the space of angular densities is used to learn about the model from data, with numerical studies showcasing its flexibility. While it is not too difficult to show that a variant of our model for the angular density is consistent under the same proviso as that of Sabourin and Naveau (2014, Sect. 3.3), as can be seen from Sect. 1 of the supplementary material, the large sample behavior of the proposed methods under a more realistic asymptotic framework remains an open question for future analysis. A comprehensive asymptotic analysis related with the current framework can be found in Padoan and Rizzelli (2022).

One could wonder why not to resort to statistical models for nonstationary extremes (e.g. Coles 2001, Sect. 6) as an alternative to methods proposed herein, as these can be used for assessing the effect of covariates on an extreme-valued response, by indexing the parameters of the GEV distribution with a covariate. Yet, since the latter models are built from the univariate theory of extremes they are not tailored for conditioning on another variable being extreme, as they fail to take on board information from the dependence structure between the extremes. Other related approaches include extremal quantile regression methods (Chernozhukov 2005), which similarly to the statistical models for nonstationary extremes, have not been designed for conditioning on another variable being extreme, as they do not take into account the dependence structure between the extremes.

While not explored here, the comparison of the fitted models for both $Y \mid X = x$ and $X \mid Y = y$, would look natural for some applied settings of interest so to get an idea of cause and effects, and indeed related ideas are analyzed by Mhalla et al. (2020). We close the paper with some comments on future research. For regressions with many predictors, it is likely that most covariates will have little effect on the response and thus one could wonder how to devise a version of the proposed method that shrinks towards zero the effect of such irrelevant covariates; the development of a Lasso (Tibshirani 1996) version of the proposed model would thus seem natural for such situation, and is left as an open problem for future research. Another natural avenue for future research would be to devise regression-type methods for exceedances on exceedances by resorting to the so-called multivariate generalized Pareto distribution (Kiriliouk et al. 2019), rather than with the multivariate extreme value distribution as herein. Additionally, the development of a version of the model that could take into account asymptotic independence by resorting to the hidden angular measure (Ramos and Ledford 2009), rather than the standard angular measure as herein, would seem natural as well. Finally, while the proposed inference methods apply to both the block maxima and threshold exceedance frameworks for multivariate extremes, if only block maxima are available there is still room for further improving the inferences of regression manifolds, and such topic might be worth further investigation and future analysis; a natural starting point would be to work directly with the likelihood of the multivariate extreme value distribution along the lines of what is discussed, for instance, in Coles (2001, Sect. 8.2.2) and Dombry et al. (2017).

### Supplementary information

Below is the link to the electronic supplementary material.

Supplementary file1 (PDF 6 MB)

## Data Availability

The datasets analysed during the current study are available from the R package DATAstudio (https://cran.r-project.org/web/packages/DATAstudio/).

## Appendix

### Appendix A. Conditional bivariate extreme value distribution

Here we derive the expression for the conditional bivariate extreme value distribution function in (10). Sklar’s theorem (Nelsen 2006, Theorem 2.3.3), implies that a joint bivariate distribution function $G:\mathbb {R}^2 \rightarrow [0,1]$ with continuous marginal distributions $G_X:\mathbb {R} \rightarrow [0,1]$ and $G_Y:\mathbb {R} \rightarrow [0,1]$ can be uniquely represented through a copula function *C* for $(x,y) \in \mathbb {R}^2$ $G(x,y) = C(G_X(x),G_Y(y)),$ or, equivalently, $C(u,v) = G(G^{-1}_X(u), G^{-1}_Y(v))$, for $(u,v) \in [0,1]^2$, where $G^{-1}_X(q) = \inf \left\{ x: G_X(x) \ge q \right\}$. Using the following well-known property of copulas,

$$\begin{aligned} C_{V\mid U}(v\mid u) :=\mathbb {P}\left( V \le v \mid U=u \right) = \frac{\partial C(u,v)}{\partial u}, \quad (u,v) \in [0,1]^2, \end{aligned}$$

we calculate the conditional distribution $(Y \mid X)$ as

$$\begin{aligned} G_{Y\mid X} (y\mid x) = C_{V\mid U}(e^{-1/y}\mid e^{-1/x}). \end{aligned}$$

In our setting

$$\begin{aligned} C(u,v)&= \exp \left[ - 2 \int _0^1 \max \{- w \log u,- (1 - w) \log v \} \, H({\mathrm {d}}w) \right] . \end{aligned}$$

Assuming *H* is absolutely continuous with density *h*, we have

$$\begin{aligned} \begin{aligned} \frac{\partial }{\partial u}&\int _0^1\max \{- w \log u,- (1 - w) \log v \} \, H({\mathrm {d}}w)\\ {}&= - \frac{\partial }{\partial u} \left( \log u \int _{\omega (u, v)}^1 w \, H({\mathrm {d}}w) + \log v \int _0^{\omega (u, v)} (1-w) \, H({\mathrm {d}}w) \right) \\ {}&= - u^{-1} \int _{\omega (u, v)}^1 w h(w) \,{\mathrm {d}}w - (\log u \log ^2 v - \log ^2 v \log u) \dfrac{u^{-1}}{\log ^3 uv} h(\omega (u, v))\\ {}&= - u^{-1} \int _{\omega (u, v)}^1 w h(w) \,{\mathrm {d}}w , \end{aligned} \end{aligned}$$

where $\omega (u, v) = \log v / \log uv$. Then, the conditional copula has the following form

$$\begin{aligned} C_{V\mid U}(v\mid u)&= 2 u^{-1} C(u,v) \int _{w(u,v)}^1 w h(w) \, {\mathrm {d}}w, \end{aligned}$$

which in turn yields

$$\begin{aligned} G_{Y|X}(y \mid x) = 2 \exp \left\{ - 2 \int _0^1 \max \left( \dfrac{w}{x}, \dfrac{1-w}{y} \right) h(w) \, {\mathrm {d}}w + x^{-1} \right\} \int _{w(x, y)}^1 w h(w) \, {\mathrm {d}}w, \quad x,y>0, \end{aligned}$$

where $$w(x,y) = x/(x+y)$$.

### Appendix B. Soft-maximum approximation for regression manifold of perfectly dependent extremes

Here we give details on the soft maximum approximation for the regression lines for perfectly dependent extremes claimed in (12). We use a smooth approximation of a maximum function called *soft-maximum*,

$$\begin{aligned} f(z_1, \dots , z_d;N) = \dfrac{1}{N}\log (e^{N z_1} + \dots + e^{N z_d}), \end{aligned}$$

which is infinitely differentiable everywhere and converges to the maximum function as $N \rightarrow \infty$ (Cook 2011). Then, the approximation of a multivariate GEV distribution function for the case of perfect dependent extremes, $G(\mathbf {x},y) = \max \{x_1^{-1}, \dots , x_p^{-1}, y^{-1}\}$ is

$$\begin{aligned} \tilde{G}(\mathbf {x}, y;N) &= \exp \left\{ - \dfrac{1}{N}\log ( e^{N y^{-1}} + e^{N x_1^{-1}} + \dots + e^{N x_p^{-1}}) \right\} \\&= ( e^{N y^{-1}} + e^{N x_1^{-1}} + \dots + e^{N x_p^{-1}})^{-1/N}, \end{aligned}$$

and its partial derivative of order *d* is

$$\begin{aligned} \begin{aligned} \tilde{g}(\mathbf {x}, y;N)&= \dfrac{\partial ^d}{\partial x_1 \cdots \partial x_p \partial y} \tilde{G}(\mathbf {x}, y;N)\\ {}&= y^{-2} \prod \limits _{i=1}^p (1 + iN) x_i^{-2} \exp \left( Ny^{-1} + \sum \limits _{i=1}^p x_i^{-1} \right) ( e^{N y^{-1}} + e^{N x_1^{-1}} + \dots + e^{N x_p^{-1}} )^{-1/N - d}. \end{aligned} \end{aligned}$$

This yields the following approximation of the conditional multivariate GEV density for perfectly dependent extremes,

$$\begin{aligned} \begin{aligned} \tilde{g}_{Y\mid \mathbf {X}} (y \mid \mathbf {x}; N)&= \dfrac{y^{-2} \prod \limits _{i=1}^p (1 + iN) x_i^{-2} \exp \left( Ny^{-1} + \sum \limits _{i=1}^p x_i^{-1} \right) ( e^{N y^{-1}} + e^{N x_1^{-1}} + \dots + e^{N x_p^{-1}})^{-1/N - d}}{\prod \limits _{i=1}^{p-1} (1 + iN) \prod \limits _{i=1}^{p} x_i^{-2} \exp \left( \sum \limits _{i=1}^p x_i^{-1} \right) ( e^{N x_1^{-1}} + \dots + e^{N x_p^{-1}})^{-1/N - p}}\\ {}&= (1+pN) y^{-2} e^{Ny^{-1}} \frac{( e^{N y^{-1}} + e^{N x_1^{-1}} + \dots + e^{N x_p^{-1}})^{-1/N - d} }{( e^{N x_1^{-1}} \dots + e^{N x_p^{-1}})^{-1/N - p}}, \end{aligned} \end{aligned}$$

and the following approximation for the corresponding conditional cumulative distribution function

$$\begin{aligned} \tilde{G}_{Y\mid \mathbf {X}} (y \mid \mathbf {x} ; N)&= \int _0^y \tilde{g}_{Y\mid \mathbf {x}} (z \mid \mathbf {x}) \, {\mathrm {d}}z\\&\begin{aligned}=&(1+pN) ( e^{N x_1^{-1}} + \dots + e^{N x_p^{-1}} )^{1/N + p}\\&\int _0^y z^{-2} e^{Nz^{-1}} ( e^{N z^{-1}} + e^{N x_1^{-1}} + \dots + e^{N x_p^{-1}})^{-1/N - d} \, {\mathrm {d}}z\end{aligned}\\&\begin{aligned}= &\dfrac{(1+pN)(-1/N)}{-1/N - d + 1} ( e^{N x_1^{-1}} + \dots + e^{N x_p^{-1}} )^{1/N + p}\\&( e^{N y^{-1}} + e^{N x_1^{-1}} + \dots + e^{N x_p^{-1}} )^{-1/N - d + 1}\end{aligned}\\ {}&=\left( \dfrac{ e^{N y^{-1}} + e^{N x_1^{-1}} + \dots + e^{N x_p^{-1}} }{e^{N x_1^{-1}} + \dots + e^{N x_p^{-1}}} \right) ^{-1/N - p}. \end{aligned}$$

Passing the last expression to the limit as $N \rightarrow \infty$ provides an ansatz for the true conditional distribution function

$$\begin{aligned} \tilde{G}_{Y\mid \mathbf {X}}(y\mid \mathbf {x}) = {\left\{ \begin{array}{ll} 1, &{} y \ge \min (x_1,\dots ,x_p)\\ 0, &{} y < \min (x_1,\dots ,x_p) \end{array}\right. } \end{aligned}$$

with $x_1,\dots ,x_p, y > 0$, from where (12) follows.

### Appendix C. Exact and limiting regression manifolds for logistic model

Here we give details on how the exact (15) and approximated (14) regression manifolds for the logistic model can be derived. The derivations below require the use of Lampert *W* function (Borwein and Lindstrom 2016) on which some properties and details can be found in the supplementary material.

#### C.1. Exact regression manifold

Here we compute the conditional quantiles for bivariate extreme value distribution and their linear approximation for large *x*. Using (10), we calculate the conditional distribution function for the logistic model; for $(x,y)\in (0,\infty )^2$, it follows that

$$\begin{aligned} \begin{aligned} G_{Y|X}(y \mid x)&= G(x,y) (x^{-1/\alpha } + y^{-1/\alpha })^{\alpha -1} x^{-1/\alpha - 1} x^2 \exp (x^{-1})\\ {}&= \exp \{ - ( x^{-1/\alpha } + y^{-1/\alpha })^{\alpha } + x^{-1} \} (x^{-1/\alpha } + y^{-1/\alpha })^{\alpha -1} x^{1-1/\alpha }. \end{aligned} \end{aligned}$$

The conditional quantiles behave differently depending on the strength of dependence between extremes. The special case of the logistic model is for $\alpha =1$, corresponding to independence between extremes for which the family of regression lines are known to be given by (11). We now derive conditional quantiles for bivariate dependent extremes, i.e. when $\alpha \in [0,1)$. Since $y \mapsto G_{Y|X}$ is continuous, a conditional quantile is a solution to

22 $$\begin{aligned} \exp \{ - ( x^{-1/\alpha } + y^{-1/\alpha })^{\alpha } \} ( x^{-1/\alpha } + y^{-1/\alpha })^{\alpha - 1} x^{1-1/\alpha }\exp (x^{-1}) = q, \end{aligned}$$

which can be written in terms of the Lambert *W* function. Rewriting (22) as

$$\begin{aligned}&\exp \left\{ - \dfrac{\alpha }{\alpha -1} ( x^{-1/\alpha } + y^{-1/\alpha })^{\alpha } \right\} ( x^{-1/\alpha } + y^{-1/\alpha })^{(\alpha - 1){\alpha }/{(\alpha -1)}} x^{{(\alpha - 1)}/{\alpha } {\alpha }/{(\alpha -1)}} \\& \times \exp \left\{ \dfrac{\alpha }{\alpha -1} x^{-1} \right\} = q^{{\alpha }/{(\alpha -1)}}\\ \Leftrightarrow&\exp \left\{ \dfrac{\alpha }{1-\alpha } ( x^{-1/\alpha } + y^{-1/\alpha } )^{\alpha } \right\} \dfrac{\alpha }{1-\alpha } ( x^{-1/\alpha } + y^{-1/\alpha })^{\alpha } = \dfrac{\alpha }{1-\alpha } x^{-1} \exp \left\{ \dfrac{\alpha }{1-\alpha } x^{-1} \right\} q^{{\alpha }/(\alpha -1)}\\ \Leftrightarrow&\dfrac{\alpha }{1-\alpha } ( x^{-1/\alpha } + y^{-1/\alpha })^{\alpha } = W \left( \dfrac{\alpha }{1-\alpha } x^{-1} e^{\alpha /(1-\alpha ) x^{-1}} q^{{\alpha }/(\alpha -1)} \right) , \end{aligned}$$

gives

23 $$\begin{aligned} y_{q\mid x} = \left[ \left\{ \dfrac{1-\alpha }{\alpha } x W \left( \dfrac{\alpha }{1-\alpha } x^{-1} e^{ \alpha /(1-\alpha ) x^{-1}} q^{{\alpha }/(\alpha -1)} \right) \right\} ^{1/\alpha } - 1 \right] ^{-\alpha } x , \quad x > 0. \end{aligned}$$

From properties of the Lambert *W* function (see supplementary material for details) it follows that

$$\begin{aligned} \underset{x \rightarrow \infty }{\lim } x \, W \left( \dfrac{\alpha }{1-\alpha } \, x^{-1} \, e^{ {\alpha }/(1-\alpha ) x^{-1}} q^{{\alpha }/{(\alpha -1)}} \right) = \dfrac{\alpha }{1-\alpha }q^{{\alpha }/{(\alpha -1)}}, \end{aligned}$$

and that the conditional quantiles tend to infinity as $x \rightarrow \infty$.

#### C.2. Limiting regression manifold

Below we show that (14) holds. To find $\gamma _q$ and $\beta _q$ we use the expansion of the principal branch of the Lambert *W* function, a solution to $z = w e^w$ when $z>0$, around 0, that is

$$\begin{aligned} W(z) = \sum \limits _{n=1}^{\infty } \dfrac{(-n)^{n-1}}{n!} z^n = z + O(z^2), \end{aligned}$$

where *O*(*z*) is big-*O* of *z*. Substituting in (23) the expansion of *W* leads to

$$\begin{aligned} \begin{aligned} y_{q\mid x}&= \bigg [ \bigg \{ \dfrac{1-\alpha }{\alpha }x \bigg ( \dfrac{\alpha }{1-\alpha } x^{-1} e^{{\alpha }/(1-\alpha ) x^{-1}} q^{{\alpha }/(\alpha -1)} + O(x^{-2} e^{{2 \alpha }/{(1 - \alpha )} x^{-1}}) \bigg ) \bigg \}^{1/\alpha } - 1 \bigg ]^{-\alpha } x\\ {}&= \bigg [ \bigg \{ e^{\alpha /(1-\alpha ) x^{-1}} q^{{\alpha }/(\alpha -1)} + O(x^{-1} e^{{2 \alpha }/{(1 - \alpha )} x^{-1}}) \bigg \}^{1/\alpha } - 1 \bigg ]^{-\alpha } x, \end{aligned} \end{aligned}$$

and taking $x \rightarrow \infty$ we find the linear asymptote of $x \mapsto y_{q\mid x}$ which is based on

24 $$\begin{aligned} \beta _q&= \lim \limits _{x \rightarrow \infty } \dfrac{y_{q \mid x}}{x} = \{ q^{-1/(1-\alpha )} - 1 \}^{-\alpha }, \end{aligned}$$

and, the more involved calculation of $\gamma _q$. The derivative of the asymptotic expansion of a function does not necessarily correspond to the asymptotic expansion of the derivative of the function; hence, we use L’Hospital with (23) and only then inject the asymptotic expansions. We have then after some tedious derivations that

25 $$\begin{aligned} \gamma _q&= \lim \limits _{x \rightarrow \infty } \left( y_{q \mid x} - \beta _q x \right) = \dfrac{\alpha }{1-\alpha } \{ q^{{1}/{(\alpha -1)}} - 1 \}^{-\alpha -1} \{ q^{{\alpha }/{(1-\alpha )}} - 1 \} q^{1 / (\alpha -1)}. \end{aligned}$$

The resulting linear approximation is as follows, for $q \in (0,1)$ and $x \gg 1$:

$$\begin{aligned} \tilde{y}_{q \mid x} = \dfrac{\alpha }{1-\alpha } \{ q^{{1}/{(\alpha -1)}} - 1 \}^{-\alpha -1} \{ q^{{\alpha }/{(1-\alpha )}} - 1 \} q^{{1}/{(\alpha -1)}} + \{ q^{-{1}/{(1-\alpha )}} - 1 \}^{-\alpha }x =\alpha _q + \beta _q x. \end{aligned}$$
