# Supplementary material for: Regression-type analysis for multivariate extreme values
Source: Extremes (Boston). 2022 Oct 21;25(4):595–622. doi: 10.1007/s10687-022-00446-6 (PMC9589765; doi:10.1007/s10687-022-00446-6)
Supplement: Supplementary file 1 — Supplementary file1 (PDF 6 MB) [file 10687_2022_446_MOESM1_ESM.pdf]

Noname manuscript No.
(will be inserted by the editor)

Regression-type analysis for multivariate extreme values: Supplementary materials

Miguel de Carvalho · Alina Kumukova · Gonçalo dos Reis

Received: date / Accepted: date
© The Author(s) 2022

1 Selected comments on Bayesian asymptotics

Since we learn about the regression manifold via a random Bernstein angular density, below we sketch some details on posterior consistency of the latter. To be precise, the result below holds for a variant of the approach from Section 3 where a prior is additionally assigned to J. The proof of consistency of random Bernstein angular densities to be presented below follows closely the line of attack of Petrone and Wasserman (2002, Theorem 2) and it resorts to a celebrated result by Schwartz (1965, Theorem 6.1). For an introduction to consistency within a Bayesian framework as well as to Schwartz theorem see, for instance, Walker (2004) and Ghosal and Van der Vaart (2015, Chapter 6).

Before introducing the key result of this section we lay the groundwork. Let $\mathscr{H}$ be the space of all probability measures over Δ_d that have a bounded, continuous density h, and that verify the moment constraint. Let Π be a prior measure over $\mathscr{H}$, and define the sequence posterior distributions

$$\Pi_n(A) = \frac{\int_A L_n(h)\,\Pi(\mathrm{d}h)}{\int_{\mathscr{H}} L_n(h)\,\Pi(\mathrm{d}h)},$$

based on a pseudo-sample of size $k := k_n = o(n)$ from $h(\mathbf{w})$, $\mathbf{W}_1, \dots, \mathbf{W}_k$, and where $L_n(h) = \prod_{i=1}^{k_n} h(\mathbf{W}_i)$. The posterior Π_n is said to be consistent at $H_0 \in \mathscr{H}$ if

$$\Pi_n(U^c) \to 0, \qquad \text{a.s. } [H_0],$$

as $n \to \infty$, for every neighborhood U of h_0, where $U^c = \mathscr{H} \backslash U$. Roughly speaking, Schwartz theorem will allow us to establish that the posterior of interest is consistent at H_0 provided that the prior assigns positive mass to every Kullback–Leibler neighborhood of h_0. In other words, the focus of the argument is to check whether $\{h : K(h_0, h) < \varepsilon\} > 0$ for every $\varepsilon > 0$, where

$$K(h_0, h) = \int_{\Delta_d} h_0(\mathbf{w}) \log\left\{\frac{h_0(\mathbf{w})}{h(\mathbf{w})}\right\} \mathrm{d}\mathbf{w}, \tag{1.1}$$

which is the same saying that we will have to check if h_0 is in the Kullback–Leibler support of Π.

As it becomes evident from the context discussed above, here we consider the same asymptotic setup as that of Sabourin and Naveau (2014, Section 3.3), that is we will analyze the large

M. de Carvalho
School of Mathematics, University of Edinburgh, The King's Buildings, Edinburgh, EH9 3FD, UK
Tel.: +44-131-650-5054
E-mail: Miguel.deCarvalho@ed.ac.uk

A. Kumukova
Maxwell Institute for Mathematical Sciences School of Mathematics, University of Edinburgh, Edinburgh UK

G. dos Reis
School of Mathematics, University of Edinburgh, The King's Buildings, Edinburgh, EH9 3FD, UK, and
Centro de Matemática e Aplicações (CMA), FCT, UNL, Quinta da Torre, 2829–516 Caparica, Portugal

sample behavior of the posterior given $\mathbf{W}_1, \ldots, \mathbf{W}_k$ independent and identically distributed observations sampled from an angular density h_0. This means that the asymptotic setup examined here should be regarded as a major simplification, in the sense that in practice margins have to be estimated and the angular density is a limiting object. Still, we believe that in a similar spirit as Sabourin and Naveau, the asymptotic analysis provided below reveals already some interesting insights on the large sample behavior under a Bayesian setup of the methods devised herein.

In what follows the prior probability mass function for J is denoted by $p(J)$, whereas $p(\pi_{\boldsymbol{\alpha}} : \boldsymbol{\alpha} \in \mathscr{F})$ is the prior density for the $m-d$ free parameters, which assumed to be induced by the prior density for all m parameters, the latter being denoted by $p(\pi_{\boldsymbol{\alpha}} : |\boldsymbol{\alpha}| = J)$.

Theorem 1 *Suppose that $H_0 \in \mathscr{H}$. In addition, suppose that $p(J) > 0$ for all $J \in \mathbb{N}$ and that*

$$g_J(\mathbf{u}) := p(\pi_{\boldsymbol{\alpha}} : \boldsymbol{\alpha} \in \mathscr{F}) \propto p(\pi_{\boldsymbol{\alpha}} : |\boldsymbol{\alpha}| = J)\, I\left(\sum_{i=1}^{J-d+1} i \sum_{|\boldsymbol{\alpha}|=J, \alpha_j = i} \pi_{\boldsymbol{\alpha}} = \frac{J}{d}\right), \qquad \mathbf{u} = (u_1, \ldots, u_{m-d}),$$

is positive over Δ_{m-d}, and it is the density of an absolutely continuous distribution function with respect to Lebesgue measure in $m-d$ dimensions. Then, the random Bernstein angular density is weakly consistent at H_0.

Proof The strategy of the proof is similar to that of Petrone and Wasserman (2002). First note that,

$$\lim_{J\to\infty} \int_{\Delta_d} \log\left[\frac{h_0(\mathbf{w})}{b\{\mathbf{w}; J, \boldsymbol{\pi}_J(h_0)\}}\right] h_0(\mathbf{w})\, \mathrm{d}\mathbf{w} = \int_{\Delta_d} \lim_{J\to\infty} \log\left[\frac{h_0(\mathbf{w})}{b\{\mathbf{w}; J, \boldsymbol{\pi}_J(h_0)\}}\right] h_0(\mathbf{w})\, \mathrm{d}\mathbf{w} = 0, \tag{1.2}$$

where $\boldsymbol{\pi}_J(h_0) = (\pi(h_0) : |\boldsymbol{\alpha}| = J)$, with

$$\pi_{\boldsymbol{\alpha}}(h_0) = H_0\left\{\left(\frac{\alpha_1 - 1}{J-d+1}, \frac{\alpha_1}{J-d+1}\right] \times \cdots \times \left(\frac{\alpha_{d-1} - 1}{J-d+1}, \frac{\alpha_{d-1}}{J-d+1}\right]\right\}.$$

Equation (1.2) follows from the uniform approximation of multivariate Bernstein polynomials (Barrientos et al. 2015, Section 4.1), and the assumption that $h_0(\mathbf{w})$ is bounded away from zero; indeed, together these imply that there exists $J_0 \in \mathbb{N}$ such that $b\{\mathbf{w}; J, \boldsymbol{\pi}(h_0)\}$ is bounded and bounded away from zero, for any $J \geq J_0$, and thus

$$\left|\log\left[\frac{h_0(\mathbf{w})}{b\{\mathbf{w}; J, \boldsymbol{\pi}_J(h_0)\}}\right]\right| < M,$$

for any $J \geq J_0$. The upshot of (1.2) is that for any $\varepsilon > 0$ there exists J_0 such that $K(h_0, b_0) < \varepsilon$, with $b_0(\cdot) := b\{\cdot; J_0, \boldsymbol{\pi}_{J_0}(h_0)\}$. Next, we will show that for any $\varepsilon > 0$, there exists $\delta > 0$ such that

$$K(h_0, b_0^*) < \varepsilon, \qquad \text{whenever } \boldsymbol{\pi}_{J_0} \in N_\delta, \tag{1.3}$$

with $N_\delta = \{\boldsymbol{\pi}_{J_0} : \max_{|\boldsymbol{\alpha}|=J_0} |\pi_{\boldsymbol{\alpha}} - \pi_{\boldsymbol{\alpha}}(h_0)| < \delta\}$ and $b_0^*(\cdot) := b(\cdot, J_0, \boldsymbol{\pi}_{J_0})$. To see this, note first that

$$\begin{aligned}\sup_{\mathbf{w}\in\Delta_d} |b_0^*(\mathbf{w}) - b_0(\mathbf{w})| &\leq \sup_{\mathbf{w}\in\Delta_d} \left\{\sum_{|\boldsymbol{\alpha}|=J_0} |\pi_{\boldsymbol{\alpha}} - \pi_{\boldsymbol{\alpha}}(h_0)|\, \mathrm{dir}_d(\mathbf{w}; \boldsymbol{\alpha})\right\} \\ &\leq (J_0 - 1) \times \cdots \times (J_0 - d + 1) \max_{|\boldsymbol{\alpha}|=J_0} |\pi_{\boldsymbol{\alpha}} - \pi_{\boldsymbol{\alpha}}(h_0)|,\end{aligned}$$

as $\mathrm{dir}_d(\mathbf{w}; \boldsymbol{\alpha}) \leq (J-1) \times \cdots \times (J-d+1)$, for all $\mathbf{w}$ and all $\boldsymbol{\alpha} \in \mathbb{N}^d$ such that $|\boldsymbol{\alpha}| = J \geq d$. Thus, there exists $\delta > 0$ sufficiently small such that $b_0^*(\mathbf{w})$ is bounded and bounded away from zero, for any $\boldsymbol{\pi}_{J_0} \in N_\varepsilon$, and hence

$$\left|\log\left\{\frac{h_0(\mathbf{w})}{b_0^*(\mathbf{w})}\right\}\right| < M$$

for all $\mathbf{w} \in \Delta_d$ and $\boldsymbol{\pi}_{J_0} \in N_\delta$. This proves (1.3). We are now ready to claim that

$$\begin{aligned}\Pi\{b : K(h_0, b) \leq \varepsilon\} &\geq \Pi\{b(\cdot, J_0, \boldsymbol{\pi}_{J_0}) : \boldsymbol{\pi}_{J_0} \in N_\delta\} \\ &\geq \Pi\{b(\cdot, J_0, \boldsymbol{\pi}_{J_0}) : \boldsymbol{\pi}_{J_0} \in N_\delta \cap A\} \\ &= p(J_0) \int_{N_\delta \cap A} g_{J_0}(\mathbf{u})\, \mathrm{d}\mathbf{u} > 0,\end{aligned}$$

where $A = \{\boldsymbol{\pi}_{J_0} : \sum_{i=1}^{J_0-d+1} i \sum_{|\boldsymbol{\alpha}|=J_0, \alpha_j=i} \pi_{\boldsymbol{\alpha}} = J_0/d\}$, given that $p(J_0) > 0$, $N_\delta \cap A$ has positive Lebesgue measure in $m-d$ dimensions, and $g_{J_0}(\mathbf{u}) > 0$ for every $\mathbf{u} \in \varDelta_{m-d}$. The final result is now a trivial consequence of Schwartz theorem. □

Theorem 1 warrants some remarks. The assumption that h_0 is bounded away from zero (i.e., $\inf_{\mathbf{w} \in \Delta_d} h_0(\mathbf{w}) > 0$) that is made in Proposition 1 can be easily relaxed using a similar argument as in Petrone and Wasserman (2002, pp. 84–85). The proof of Proposition 1 uses the fact that $\mathrm{dir}_d(\mathbf{w}; \boldsymbol{\alpha}) \leq (J-1) \times \cdots \times (J-d+1)$, for all $\mathbf{w}$ and all $\boldsymbol{\alpha} \in \mathbb{N}^d$ such that $|\boldsymbol{\alpha}| = J \geq d$. Such inequality trivially extends a related claim made by Petrone and Wasserman (2002) for the Beta density and for completeness we include here a proof of this result. Our proof will resort to the following Stirling double inequality

$$e^{3/2\{1-\log(3/2)\}} n^{n+1/2} e^{-n} < n! < e\, n^{n+1/2} e^{-n}, \tag{1.4}$$

which can be found in Feller (1967, Eq. (9.5)), and which holds for any $n \in \mathbb{N}$.

Lemma 1 *The density of the Dirichlet distribution obeys the following inequality,*

$$dir_d(\mathbf{w}; \boldsymbol{\alpha}) \leq b_d(\boldsymbol{\alpha}) := (J-1) \times \cdots \times (J-d+1),$$

for all $\mathbf{w} \in \Delta_d$ *and all* $\boldsymbol{\alpha} \in \mathbb{N}^d$ *such that* $|\boldsymbol{\alpha}| = J \geq d$ *with* $d \geq 2$.

Proof Suppose first that $\boldsymbol{\alpha} \in \mathbb{N}^d \backslash \{\mathbf{1}_d\}$. Then, by evaluating the density of the Dirichlet distribution at its mode it follows that

$$\begin{aligned}\mathrm{dir}_d(\mathbf{w}; \boldsymbol{\alpha}) &\leq \left[\frac{(J-1)!}{\prod_{j=1}^d \{(\alpha_j - 1)!\}}\right] \left[\frac{\prod_{j=1}^d \{(\alpha_j - 1)^{\alpha_j - 1}\}}{(J-d)^{J-d}}\right] \\ &= \left[\frac{(J-d)!}{\prod_{j=1}^d \{(\alpha_j - 1)!\}}\right] \left[\frac{\prod_{j=1}^d \{(\alpha_j - 1)^{\alpha_j - 1}\}}{(J-d)^{J-d}}\right] b_d(\boldsymbol{\alpha}) \\ &= a\, b_d(\boldsymbol{\alpha}).\end{aligned}$$

Next, we show that $a \leq 1$ from where the final result follows. Note first that,

$$\begin{aligned}a &= \left[\frac{(J-d)!}{\prod_{j=1}^d (\alpha_j - 1)!}\right] \left[\frac{\prod_{j=1}^d \{(\alpha_j - 1)^{\alpha_j - 1}\}}{(J-d)^{J-d}}\right] \\ &\leq \frac{e(J-d)^{J-d+1/2} e^{-(J-d)}}{(J-d)^{J-d}} \times \frac{\prod_{j=1}^d \{(\alpha_j - 1)^{\alpha_j - 1}\}}{\prod_{j=1}^d \{e^{3/2\{1-\log(3/2)\}} (\alpha_j - 1)^{\alpha_j - 1} e^{-(\alpha_j - 1)}\}} \\ &= \frac{\sqrt{|\boldsymbol{\alpha}| - d}}{\prod_{j=1}^d \sqrt{\alpha_j - 1}} \times \frac{e}{e^{3d/2\{1-\log(3/2)\}}} \times \frac{e^{-(J-d)}}{e^{-(|\boldsymbol{\alpha}|-d)}} \\ &\leq \frac{e\sqrt{d}}{e^{3d/2\{1-\log(3/2)\}}} := g(d),\end{aligned} \tag{1.5}$$

where the first inequality follows from Stirling inequality (1.4) and where the second inequality follows by noticing that $f(\boldsymbol{\alpha}) := \sqrt{|\boldsymbol{\alpha}| - d}/\prod_{j=1}^d \sqrt{\alpha_j - 1}$ is decreasing in α_j, and hence $f(\boldsymbol{\alpha}) \leq f(2 \times \mathbf{1}_d) = \sqrt{d}$, for any $\boldsymbol{\alpha} \in \mathbb{N}^d \backslash \{\mathbf{1}_d\}$. The fact that $a \leq 1$ now follows by observing that $g(d)$ in (1.5) is decreasing and hence $a \leq g(d) \leq g(2) \approx 0.65 \leq 1$. So far we have assumed that $\boldsymbol{\alpha} \in \mathbb{N}^d \backslash \{\mathbf{1}_d\}$, and to finish the proof we just need to consider the remainder cases for $\boldsymbol{\alpha}$. If $\boldsymbol{\alpha} = \mathbf{1}_d$, then $b_d(\boldsymbol{\alpha}) = (d-1)!$ and hence $\mathrm{dir}_d(\mathbf{w}; \mathbf{1}_d) = (d-1)! \leq b_d(\boldsymbol{\alpha})$ as required. Finally, for

the last case suppose without loss of generality that $\alpha_1 = 1$ and that $\alpha_j \geq 1$ for $j = 2, \dots, d$. Then, trivially $b_d(\mathbf{1}_d) \leq b_d\{(1, \alpha_2, \dots, \alpha_d)\}$, for $\alpha_j \geq 1$ with $j = 2, \dots, d$, and hence

$$\begin{aligned}\mathrm{dir}_d\{\mathbf{w}; (1, \alpha_2, \dots, \alpha_d)\} &= \frac{(|(1, \alpha_2, \dots, \alpha_d)| - 1)!}{\prod_{j=2}^{d}\{(\alpha_j - 1)!\}} \prod_{j=2}^{d} w_j^{\alpha_j - 1} \\ &\leq (|\mathbf{1}_d| - 1)! = (d-1)! = b_d(\mathbf{1}_d) \leq b_d\{(1, \alpha_2, \dots, \alpha_d)\},\end{aligned}$$

which finally concludes the proof. □

2 Details on the Lambert *W* function

The Lambert W function is used in the paper for deriving the regression manifold for the logistic model (see Example 1 and Appendix D), and thus we offer here some details on it. Formally, the Lambert W function is a set of functions representing the inverse relation of the function $f(z) = ze^z$ for any complex z. Since we deal only with positive real valued z, the equation $f(z) = ze^z$ has only one solution $w = W(z)$, with W being the principal branch of the Lambert W function. A useful property of this function is that for any constant $a \in \mathbb{R}$ one has

$$\lim_{z \to \infty} zW(a/z) = \lim_{z \to \infty} ae^{-W(a/z)} = a,$$

which is derived from

$$\lim_{z \to \infty} \frac{a}{z} = \lim_{z \to \infty} e^{W(a/z)} W(a/z) \quad \Rightarrow \quad \lim_{z \to \infty} W(a/z) = 0.$$

See Borwein and Lindstrom (2016) for further details.

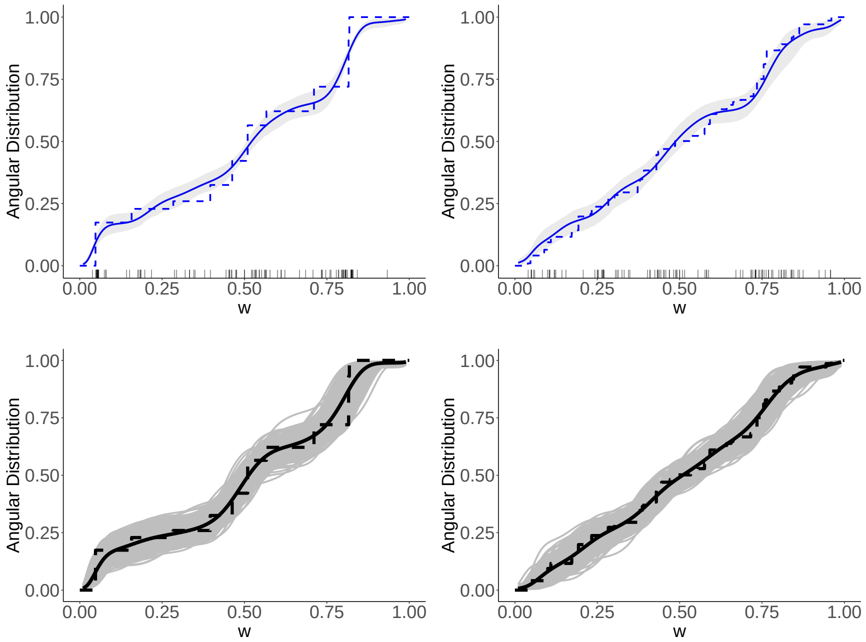

Fig. 3.1 Performance under a discrete angular measure using the scenario from Section 3.1 of the supplementary materials. Top: posterior mean angular cumulative distribution function (solid line) with 95%-credible bands (in gray) and the true angular cumulative distribution function (dashed line) for a single-run experiment with $r = 10$ and $r = 50$. Bottom: posterior mean angular cumulative distribution functions for each of 500 Monte Carlo samples (gray lines) with their mean (black line) plotted against the true cumulative distribution function (dashed line) for $r = 10$ and $r = 50$.

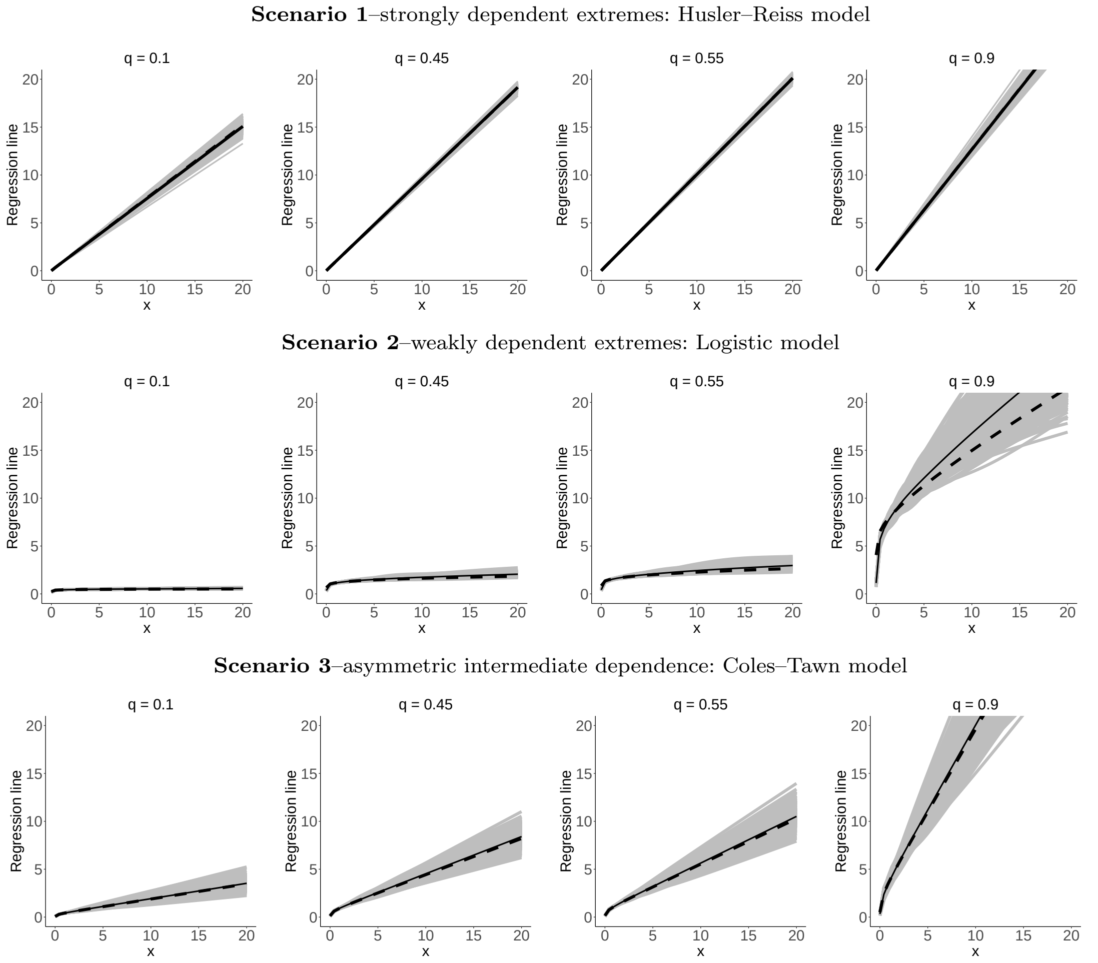

Fig. 3.2 Redoing the simulation from Section 4 of the paper with $n = 10\,000$. Posterior mean regression lines L_q for $q \in \{0.1, 0.45, 0.55, 0.9\}$ and $x \in (0, 20]$ for each of the 500 Monte Carlo samples (gray lines) plotted against the true (dashed line) for Husler–Reiss, Logistic, and Coles–Tawn bivariate extreme value models (top to bottom). The solid black line represents the Monte Carlo mean.

3 Additional numerical evidence

3.1 The case of a discrete angular measure

In this section we report the results of a further simulation scenario under a discrete angular measure. Here, we use the same MCMC configuration, prior specification, and simulation study setup as those set in Section 4 in the paper. The simulation scenario considered next is based on the max factor model of Einmahl et al. (2012, Example 2). Specifically, let $Z_1, \dots, Z_r$ be a sequence of independent unit Frechét random variables, and define the bivariate random vector

$$(X, Y) = \left(\max_{i=1,\dots,r} \{a_{i1} Z_i\}, \max_{i=1,\dots,r} \{a_{i2} Z_i\} \right),$$

where $a_{ij} \geq 0$, for any i, j, and $a_{i1} + a_{i2} > 0$, for $i = 1, \dots, r$. Then, the associated angular measure is discrete and has r atoms given by

$$\omega_i = \left(\frac{b_{i1}}{b_{i1} + b_{i2}}, \frac{b_{i2}}{b_{i1} + b_{i2}} \right), \quad i = 1, \dots, r,$$

with atom i having mass $0.5(b_{i1} + b_{i2}) > 0$, where $b_{ij} := a_{ij} / \sum_{i=1}^{r} a_{ij}$, for $j = 1, 2$. In both scenarios, the a_{ij} were fixed by sampling once from a standard exponential distribution, and we then conduct the Monte Carlo simulation given those fixed values of a_{ij}. Figure 3.1 displays the outcome of a one shot experiment along with the results from a Monte Carlo study for

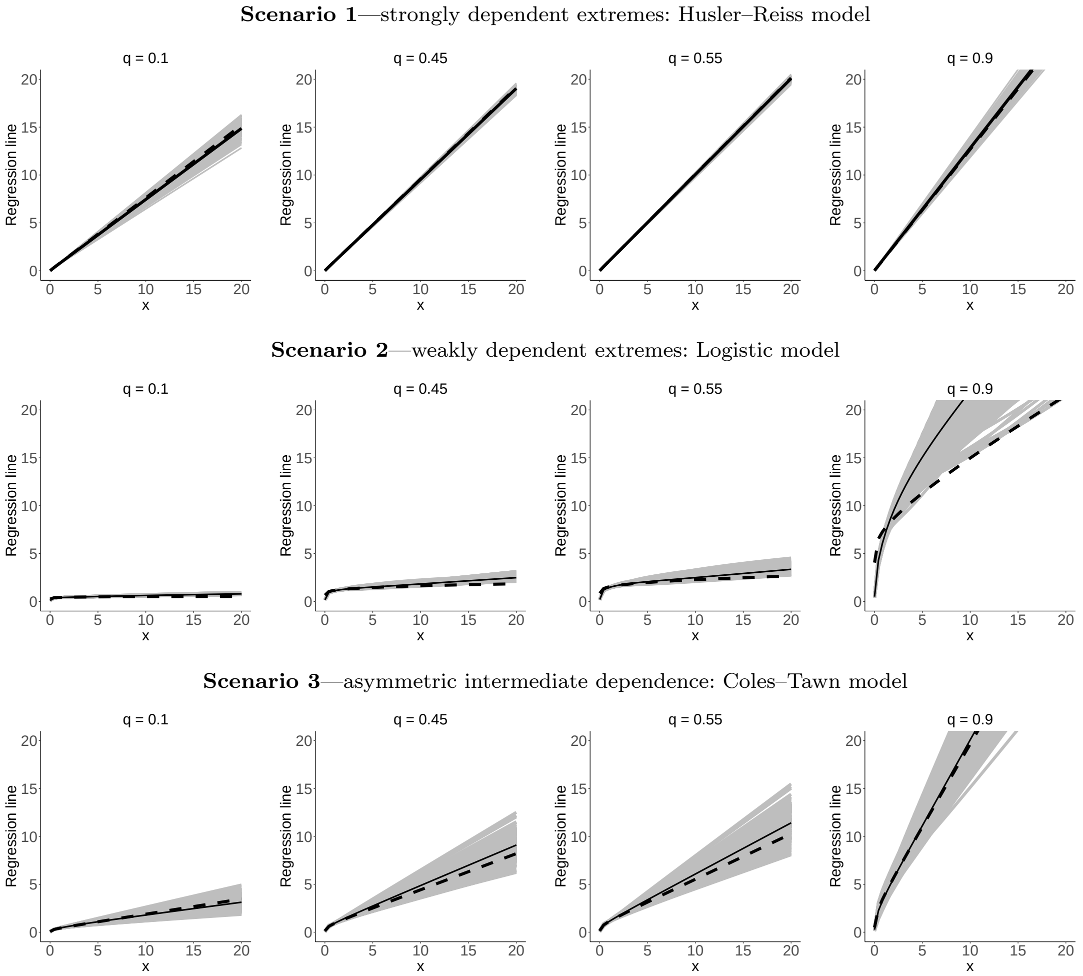

Fig. 3.3 Redoing the simulation from Section 4 of the paper using the rule of thumb of Guan (2016) for selecting J rather than the approach of Hanson et al. (2017). Posterior mean regression lines L_q for $q \in \{0.1, 0.45, 0.55, 0.9\}$ and $x \in (0, 20]$ for each of the 500 Monte Carlo samples (gray lines) plotted against the true (dashed line) for Husler–Reiss, Logistic, and Coles–Tawn bivariate extreme value models (top to bottom). The solid black line represents the Monte Carlo mean.

$r = 10$ and $r = 50$. As it can be seen from the latter chart, the fitted angular measure recovers the true target reasonably well for both cases, and as expected the performance improves with increasing r. Hence, despite the fact that our prior is defined on the space of continuous angular measures, the results indicate a satisfactory performance even for the case of a discrete angular measure.

3.2 Supporting Monte Carlo experiments

This section reports a number of supplementary Monte Carlo experiments. We have repeated the Monte Carlo simulation study from the paper by thresholding the radial component at its 95% quantile, rather than at the 98% quantile. The fits reported in Figure 3.4 are reasonably in line with those from Figure 4.3 in the paper. In addition, we have also repeated the simulation study from the paper but for $n = 10\,000$, rather than for $n = 5\,000$. Figure 3.2 depicts a moderate improvement in the fits when $n = 10\,000$ in line with the expected frequentist behavior of the proposed Bayesian methodologies. Finally, we have also re-executed the simulation study from the paper but using the approach of Guan (2016) for choosing J, rather than that of Hanson et al. (2017).

Contrarily to the approach of Hanson et al. (2017) (which only requires fitting the model once), the approach of Guan (2016) requires fitting the Bernstein polynomial model several

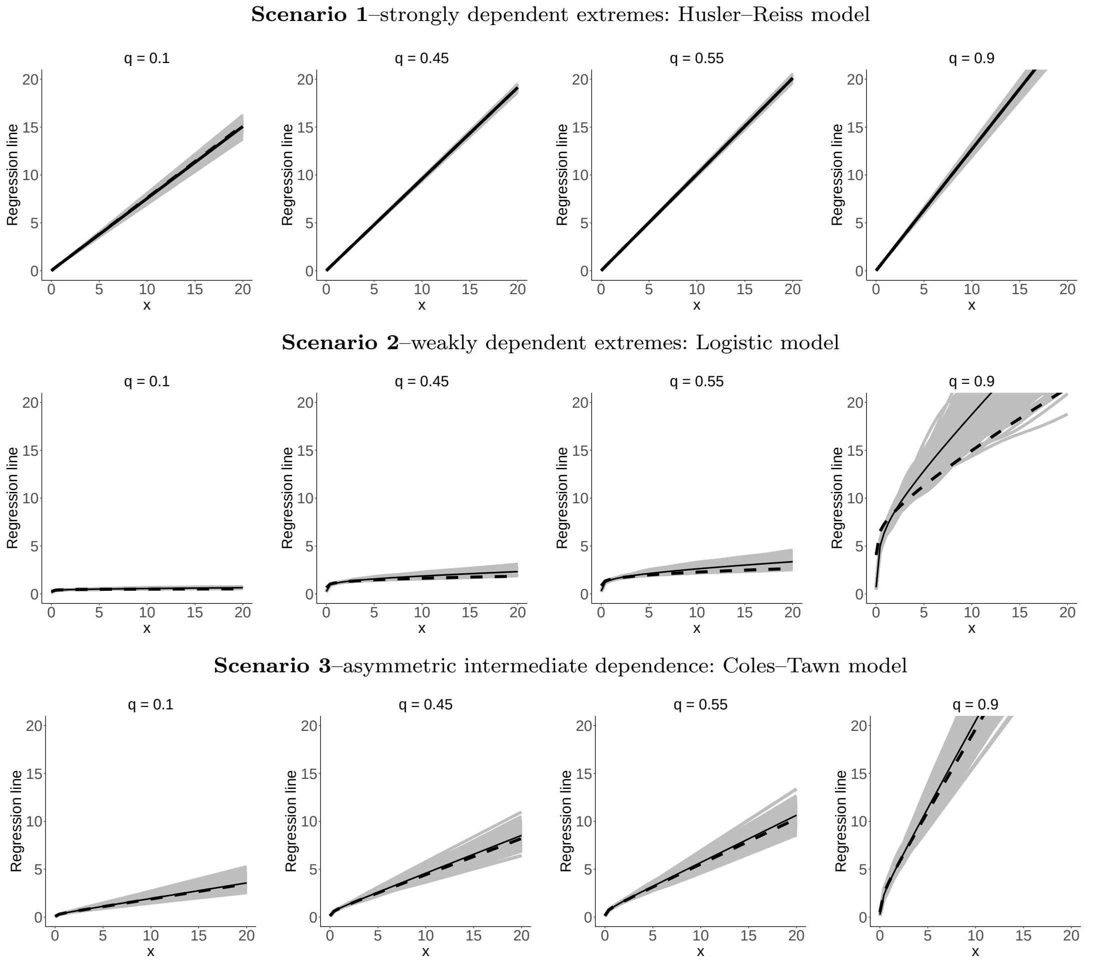

Fig. 3.4 Redoing the simulation from Section 4 of the paper by thresholding the data at the 95% quantile. Posterior mean regression lines L_q for $q \in \{0.1, 0.45, 0.55, 0.9\}$ and $x \in (0, 20]$ for each of the 500 Monte Carlo samples (gray lines) plotted against the true (dashed line) for Husler–Reiss, Logistic, and Coles–Tawn bivariate extreme value models (top to bottom). The solid black line represents the Monte Carlo mean.

times, for a sequence of values of J, and then sets the optimal J as the changepoint of the log likelihood ratio over a set of consecutive model degrees. Figure 3.3 should be compared with Figure 4.3 in the paper and it showcases that our strategy for choosing J has a comparable performance, if not superior, with respect to the rule of thumb of Guan (2016).

3.3 Induced prior for p-covariate setting

In this section we report two one-shot numerical experiments aimed at illustrating the approach in Section 3.2 in the paper, that induces a prior on the space of all regression manifolds by resorting to Bernstein polynomials and an approximation of a multivariate GEV density due to Cooley et al. (2012). For the numerical experiments in this supplementary material, we test our model by taking a trivariate logistic extreme value distribution with dependence parameter $\alpha = 0.1$ ('strongly' dependent extremes) for the case $p = 2$, i.e. with the trivariate GEV distribution

$$G(x_1, x_2, y) = \exp\{-(y^{-1/\alpha} + x_1^{-1/\alpha} + x_2^{-1/\alpha})^{\alpha}\}, \quad x_1, x_2, y > 0.$$

We generate two samples of sizes $n = 10\,000$ and $n = 20\,000$ which, after thresholding at 95% empirical quantiles of the pseudo-radius, yield $k = 500$ and $k = 1000$ data points to fit the model. Here, we use a similar prior specification and MCMC setup as in Section 4.1 of the paper.

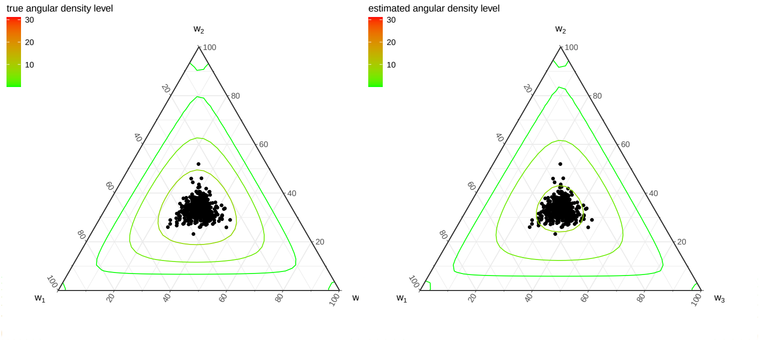

Fig. 3.5 Level plots of the true angular density (left) along with the posterior mean estimate resulting from the methods from Section 3.2 (right) on $n = 10000$ observations for the trivariate logistic extreme value distribution, on a single-run experiment, with dependence parameter $\alpha = 0.1$.

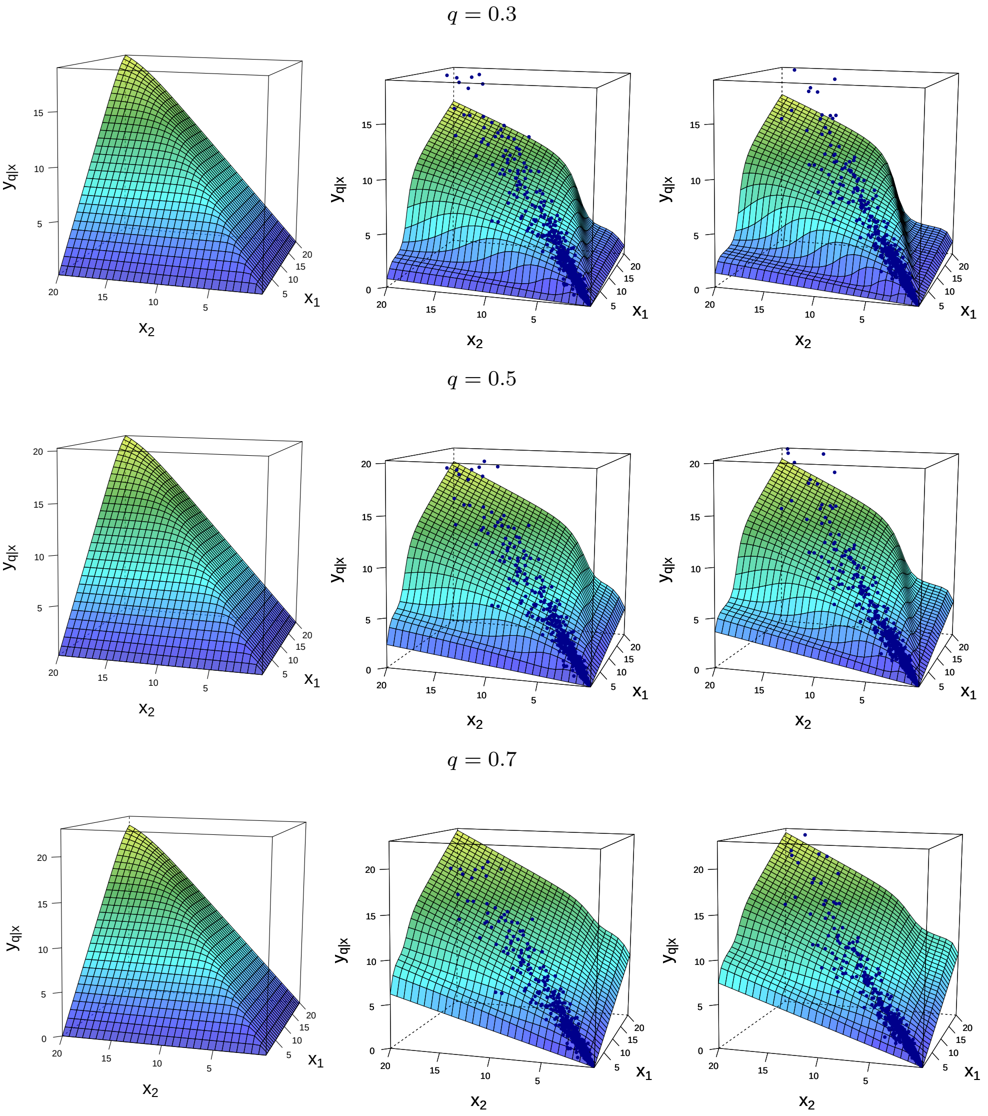

Fig. 3.6 The true L_q (left) for $q \in \{0.3, 0.5, 0.7\}$ (top to bottom) along with the posterior mean estimate resulting from the methods from Section 3.3 on $n = 10000$ (middle) and $n = 20000$ (right) observations for the trivariate logistic extreme value distribution, on a single-run experiment, with the dependence parameter $\alpha = 0.1$ over the domain $\mathbf{x} = (x_1, x_2) \in (0, 20]^2$.

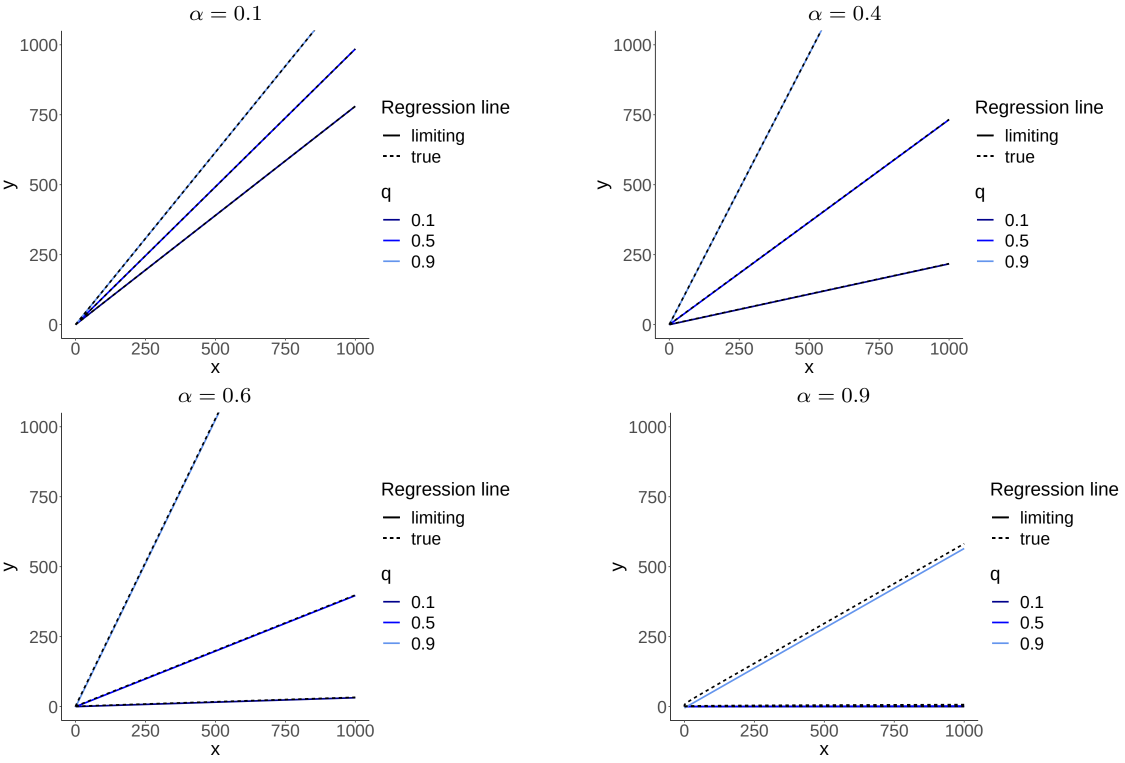

Fig. 4.1 Cross-sections of the true (black dashed line) and limiting (solid line) regression manifold for bivariate logistic model for $q \in \{0.1, 0.5, 0.9\}$.

Figure 3.5 indicates that the proposed estimator of the angular density captures reasonably well the dependence between extremes by concentrating around the barycenter of the simplex, though in a less pronounced form than the true density. As can be seen from Figure 3.6, the resulting fitted regression lines resemble the true ones, L_q, and increasing sample size improves the fit as the lateral surfaces of the estimates become more slanting, for $q \in \{0.3, 0.5, 0.7\}$.

4 Comparing exact and limiting regression manifold for logistic model

Here we illustrate how the exact and limiting regression manifold for logistic model compare; see Appendix C for details on the derivation of these. As it can be seen from Figures 4.1–4.2, the linearly approximated regression manifold derived in Appendix C.1 in the paper offers a sensible approximation of the true regression manifold, for large values of x.

5 Further empirical analysis

In this section we present results of testing multivariate regular variation applying the methods of Einmahl et al. (2021) to negative log-returns of the NYSE and NASDAQ. As can be seen from Figure 5.1, at a 5% significance level there is no evidence to reject that the pair (NYSE, NASDAQ) follows a MRV distribution for a broad range of thresholds. In addition, we also present below the reverse analysis to that presented in Section 5 of the paper; that is, here NASDAQ is the response, whereas NYSE is taken as covariate. Figure 5.3 is thus the equivalent of Fig. 5.2 in the paper but for the reverse analysis; and the same applies to Table 1, which is the reverse analysis equivalent of Table 1 in the paper. Interpretations follow along the same lines as in Section 5 of the paper.

To supplement the analysis of this extremal asymmetry from the paper we have also fitted—using our Bernstein polynomial prior—the coefficient of extremal asymmetry (Semadeni 2020)

$$\varphi = \frac{A'(1/2)}{2 - 2A(1/2)}, \tag{5.1}$$

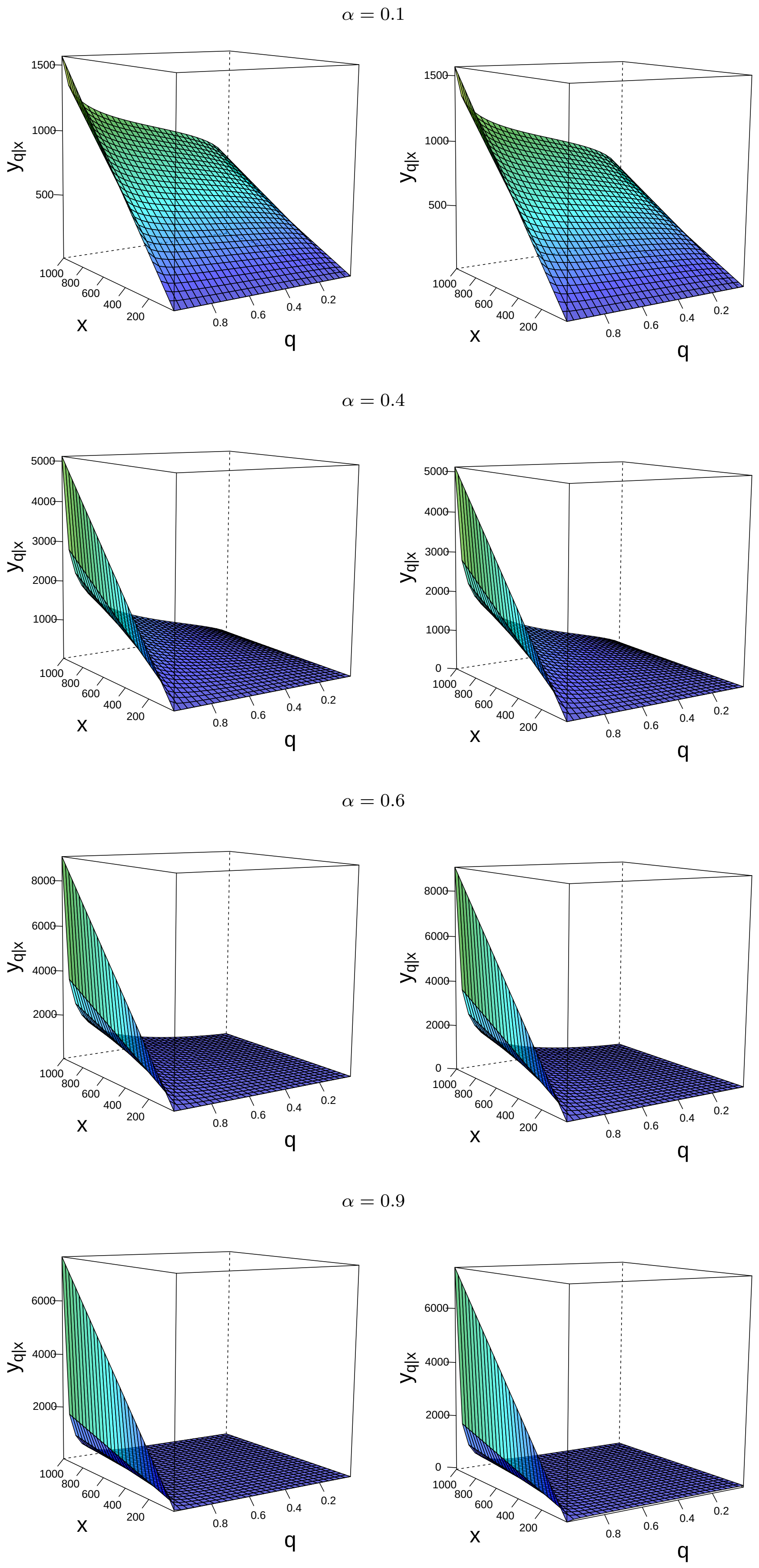

Fig. 4.2 The true (left) and limiting (right) regression manifold for bivariate logistic model in order of decreasing dependence (from top to bottom) with the dependence parameter $\alpha \in \{0.1, 0.4, 0.6, 0.9\}$.

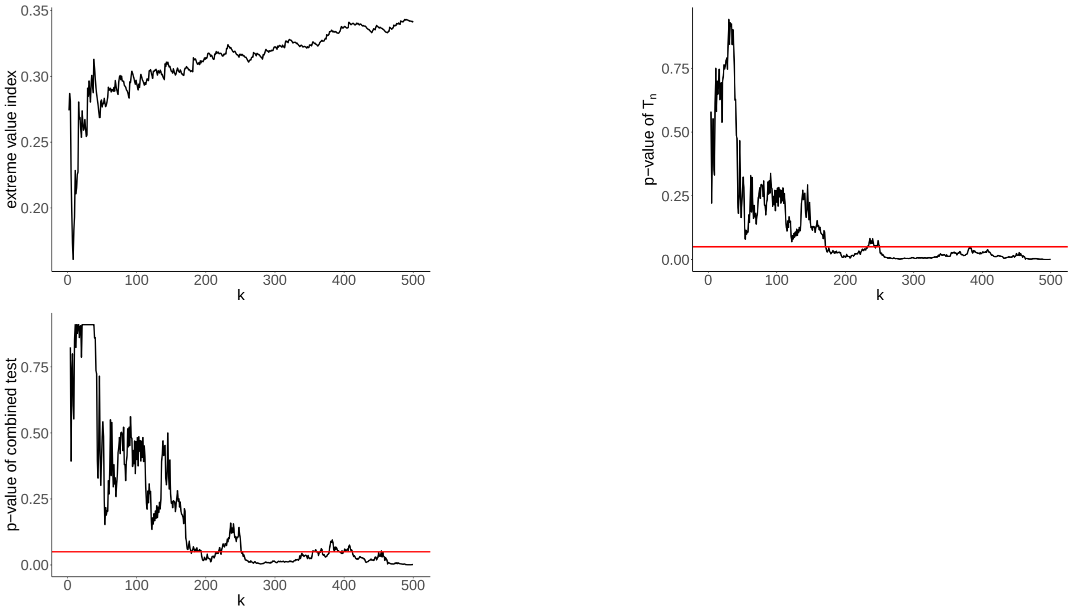

Fig. 5.1 Upper left: Hill estimates of extreme value index of the radius for different thresholds k. Upper right: p-values of T_n test for independence between the radius and pseudo-angles with the horizontal line corresponding to significance level of 5%. Lower left: p-values of combined test for the combined test for multivariate regular variation with the horizontal line corresponding to significance level of 5%.

Table 1 Predicted 75%, 90% and 95% quantiles of losses on NASDAQ evaluated for 1%, 2% and 3% weekly maxima losses on NYSE, with 95% credible intervals in brackets; negative log-returns used as proxy for losses

NASDAQ	NYSE		
	0.01	0.02	0.03
75%	0.0136	0.0249	0.0359
	(0.0128, 0.0146)	(0.0236, 0.0262)	(0.0339, 0.0375)
90%	0.0210	0.0328	0.0439
	(0.0202, 0.0224)	(0.0307, 0.0341)	(0.0413, 0.0476)
95%	0.0268	0.0389	0.0529
	(0.0254, 0.0284)	(0.0371, 0.0400)	(0.0498, 0.0570)

where A is the Pickands dependence function, that is, $A(t) = 1 - t + 2\int_0^t H(w)\mathrm{d}w$, for $t \in [0, 1]$. The obtained coefficient is 0.226, which confirms the extremal asymmetry foreseen in Figure 5.1 in the paper.

We further examine how our methods for learning about the regression manifold perform in terms of quantile verification score (QVS) of Bentzien and Friederichs (2014). Loosely speaking, the QVS is an expected quantile score which is defined by a check loss function of Koenker (2005), i.e. $\rho_\tau(u) = u\tau I(u \geq 0) + u(\tau - 1)I(u < 0)$, and essentially shows how good a quantile forecast provided by a model is (the smaller the value the better). To calculate the QVS we split our data into train (first two-thirds of observations) and test (last third of observations) sets. We learn about the regression manifold thresholding the train set data at the 95% quantile and running a MCMC of length 10 000 and a burn-in of 4 000 with other parameters being the same. Figure 5.4 depicts QVS evaluated over a grid of quantiles on the unit interval and indicates that the exact approach outperforms the approximate one, with the approximated approach resulting from the combination of our random Bernstein polynomial prior with the approximation of Cooley et al. (2012, Proposition 1).

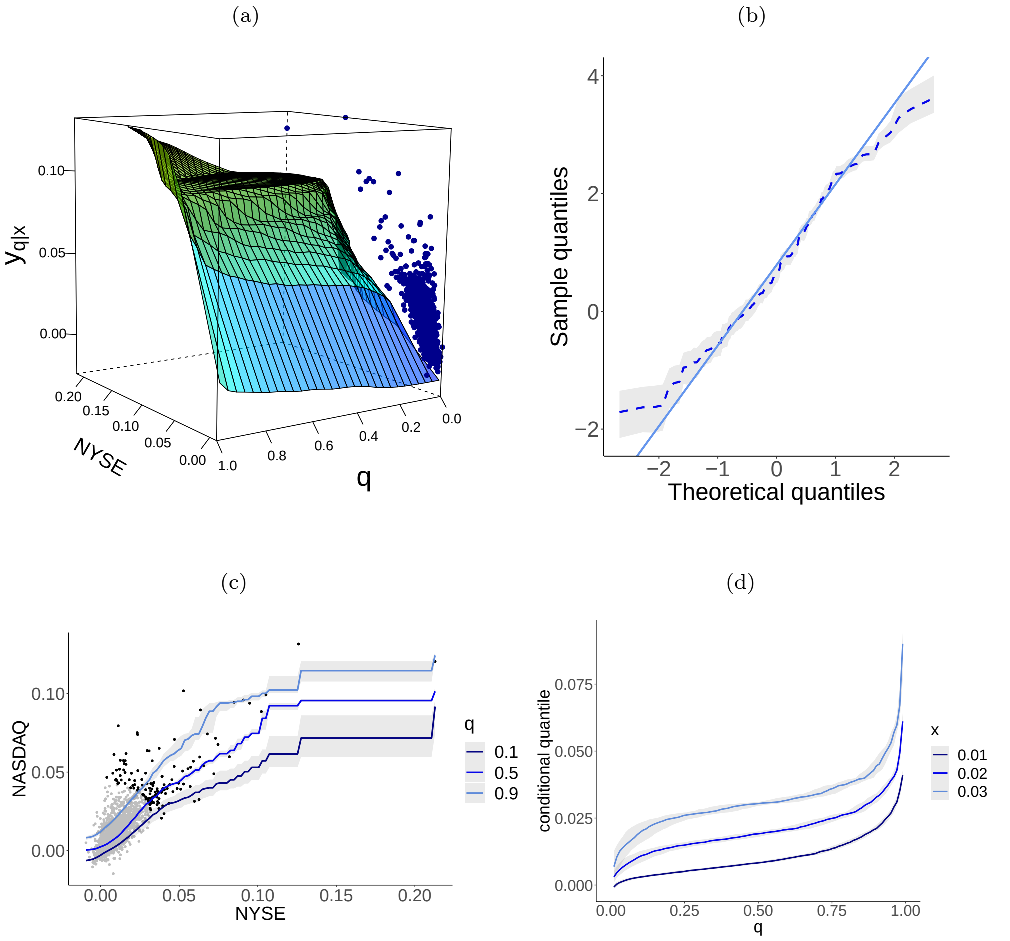

Fig. 5.2 (a) Posterior mean regression manifold $\mathscr{L}$ for NASDAQ given NYSE along with joint negative log returns overlaid on one of the faces of the box. (b) QQ-plot of randomized quantile residuals; the dashed line represents the posterior mean plotted along with credible bands. (c) Posterior mean regression lines L_q for $q \in \{0.1, 0.5, 0.9\}$ for NYSE given NASDAQ along with 95% credible bands and plotted against joint negative log returns. (d) Posterior mean conditional quantile curves $\{y_{q|x} : q \in (0, 1)\}$ of negative log returns on NASDAQ for $x \in \{0.01, 0.02, 0.03\}$, along with 95% credible bands, corresponding to negative log returns on NYSE in the original margins.

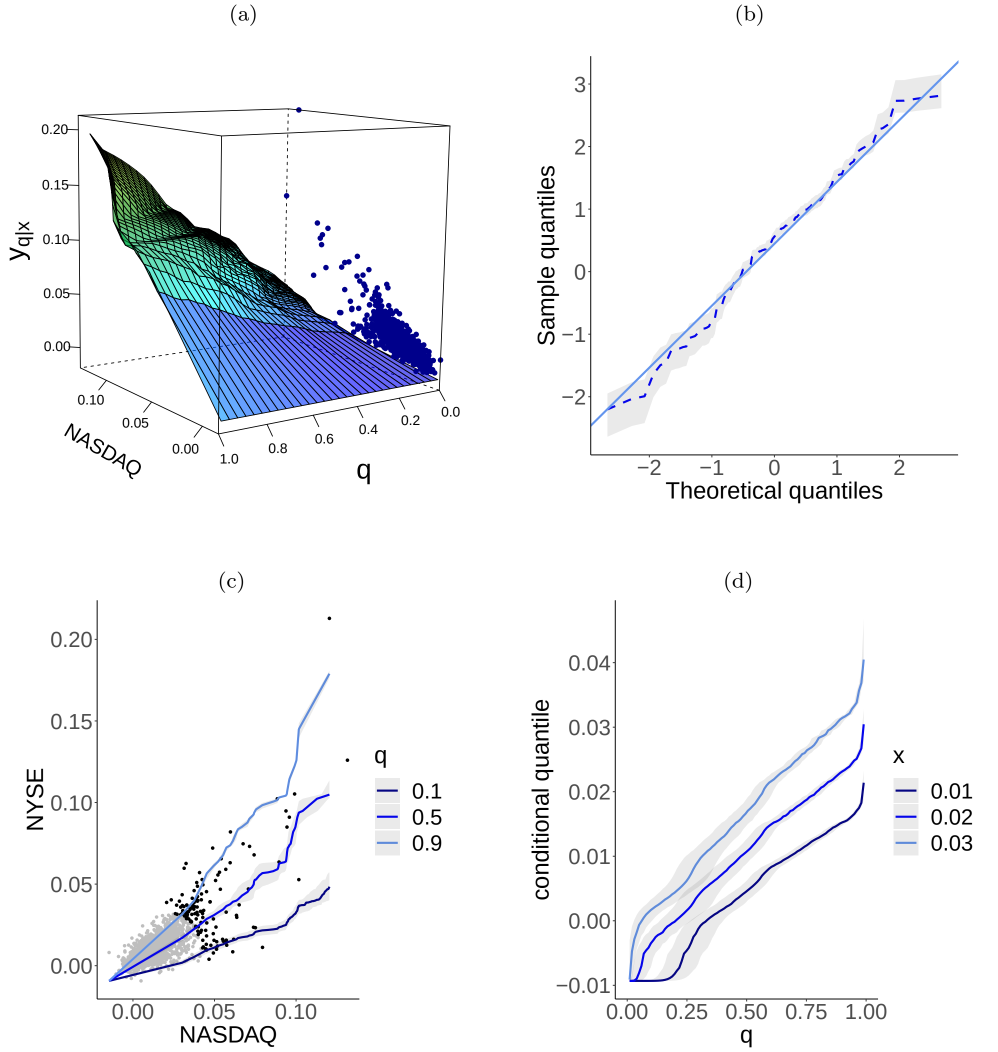

Fig. 5.3 Fit based on the approximation of Cooley et al. (2012): (a) Posterior mean regression manifold $\mathscr{L}$ for NYSE given NASDAQ along with joint negative log returns overlaid on one of the faces of the box. (b) QQ-plot of randomized quantile residuals; the dashed line represents the posterior mean plotted along with credible bands. (c) Posterior mean regression lines L_q for $q \in \{0.1, 0.5, 0.9\}$ for NYSE given NASDAQ along with 95% credible bands and plotted against joint negative log returns. (d) Posterior mean conditional quantile curves $\{y_{q|x} : q \in (0,1)\}$ of negative log returns on NYSE for $x \in \{0.01, 0.02, 0.03\}$, along with 95% credible bands, corresponding to negative log returns on NASDAQ in the original margins.

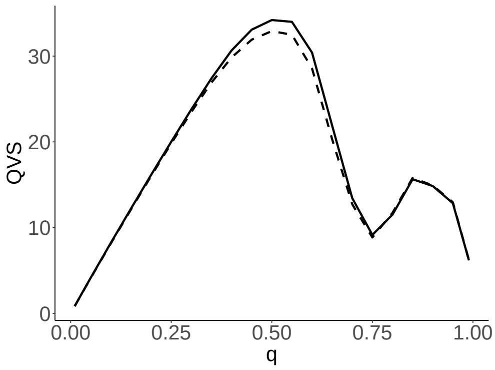

Fig. 5.4 Quantile verification scores; computed over a grid of quantiles in $(0, 1)$ for exact (dashed line) and approximate (solid line) approaches.

References

Barrientos AF, Jara A, Quintana FA (2015) Bayesian density estimation for compositional data using random Bernstein polynomials. J Stat Plann Infer 166:116–125

Bentzien S, Friederichs P (2014) Decomposition and graphical portrayal of the quantile score. Quarterly J Royal Meteo Soc 140(683):1924–1934

Borwein JM, Lindstrom SB (2016) Meetings with Lambert $\mathcal{W}$ and other special functions in optimization and analysis. Pure Appl Funct Anal 1(3):361–396

Cooley D, Davis RA, Naveau P (2012) Approximating the conditional density given large observed values via a multivariate extremes framework, with application to environmental data. Ann Appl Stat 6(4):1406–1429

Einmahl JH, Krajina A, Segers J (2012) An M-estimator for tail dependence in arbitrary dimensions. Ann Stat 40(3):1764–1793

Einmahl JH, Yang F, Zhou C (2021) Testing the multivariate regular variation model. Journal of Business & Economic Statistics 39(4):907–919

Feller W (1967) An Introduction to Probability Theory and its Applications, vol 1. Wiley, New York

Ghosal S, Van der Vaart AW (2015) Fundamentals of Nonparametric Bayesian Inference. Cambridge University Press, Cambridge

Guan Z (2016) Efficient and robust density estimation using Bernstein type polynomials. J Nonparam Stat 28(2):250–271

Hanson TE, de Carvalho M, Chen Y (2017) Bernstein polynomial angular densities of multivariate extreme value distributions. Stat Prob Lett 128:60–66

Koenker R (2005) Quantile Regression. Cambridge University Press, Cambridge, MA

Petrone S, Wasserman L (2002) Consistency of Bernstein polynomial posteriors. J Royal Stat Soc Ser B 64(1):79–100

Sabourin A, Naveau P (2014) Bayesian Dirichlet mixture model for multivariate extremes: A reparametrization. Comput Stat Data Anal 71:542–567

Schwartz L (1965) On Bayes procedures. Zeitschrift für Wahrscheinlichkeitstheorie und verwandte Gebiete 4(1):10–26

Semadeni CA (2020) Inference on the angular distribution of extremes. PhD thesis, EPFL—Swiss Federal Institute of Technology

Walker SG (2004) Modern Bayesian Asymptotics. Stat Science 19(1):111–117
